# Clinical characterization of CCT2 and its role in autophagy regulation during age-related macular degeneration

**DOI:** 10.1038/s41598-025-01907-1

**Published:** 2025-05-15

**Authors:** Lin Wang, Ling-xiao Wang, Mu-ye Li, Rong Zhang, Guo-hong Zhou

**Affiliations:** 1 Department of Ophthalmology, Shanxi Eye Hospital Affiliated to Shanxi Medical University, Taiyuan, 030002 China; 2 Department of Colorectal and Anal Surgery, Shanxi Provincial People’s Hospital (Fifth Hospital of Shanxi Medical University), Taiyuan, 030001 China

**Keywords:** Age related macular degeneration, Neurodegeneration, CCT2, Autophagy, Drusen, Computational biology and bioinformatics, Molecular biology, Biomarkers, Pathogenesis, Eye diseases

## Abstract

Age-related macular degeneration (AMD) is the leading cause of vision loss in the elderly, and the role of chaperonin containing TCP1 subunit 2 (CCT2) remains unclear. This study aims to elucidate the mechanistic link between CCT2 and AMD, contributing to improved understanding and potential therapeutic strategies. Retinal and RPE-Choroid transcriptome array data from 130 AMD patients and 121 normal donors (GSE29801 dataset) were reanalyzed to assess CCT2 expression across different AMD subtypes, age groups, and genders. Single-sample gene set enrichment analysis was performed to explore correlations with autophagy-related genes and other established AMD causes. Additionally, CCT2 expression was validated in sodium iodate (NaIO₃)-induced 661 W cells (photoreceptor-like cells) using quantitative real-time PCR (qRT-PCR). CCT2 was significantly enriched in advanced AMD retinas compared to intermediate stages in retina (both macular and extramacular) and early stages in extramacular retina (*p* < 0.05). NaIO_3_-treated 661 W cells exhibited a similar expression trend, confirming transcriptomic findings. CCT2 is significantly upregulated in advanced AMD and may contribute to drusen degradation. It shows potential as both a biomarker and an independent diagnostic indicator, particularly for advanced-stage AMD.

**Supplementary Information:**

The online version contains supplementary material available at 10.1038/s41598-025-01907-1.

## Introduction

Age-related Macular Degeneration (AMD) is a progressive eye disorder that notably deteriorates central vision. This condition arises from a combination of pathological findings and is recognized as a foremost cause of vision loss within the elderly community, especially in developed nations^1^. In China, the prevalence of early AMD ranged from 1.79 to 10.05%, and for advanced AMD, it varies from 0.38 to 3.88%^2^. As China grapples with an escalating aging population, the incidence of AMD is notably increasing. This surge not only profoundly impacts the quality of life for patients but also induces significant economic strain. The worldwide cost associated with visual impairment due to AMD is projected to be approximately $343 billion, with direct healthcare expenditures constituting a staggering $255 billion^3^.

Clinically, AMD is categorized into early, intermediate, and advanced stages based on disease progression^4^. Advanced AMD is further divided into geographic atrophy (GA) and neovascular AMD^4^. It may involve focal areas of RPE degeneration and photoreceptor loss (referred to as ‘GA’) or neovascular changes (such as choroidal neovascular membrane (CNV), subretinal or sub-RPE hemorrhage), as well as subretinal fibrosis^5^. Despite the effectiveness of intravitreal anti-VEGF injections in curbing the progression of some neovascular AMD, treatment options for AMD remain largely limited, finding a cure remains a significant challenge in the field^6^. Indeed, our limited comprehension of AMD’s genesis obstruct the formulation of efficacious prevention and treatment tactics^7^. Etiologic treatment targeting the early or intermediate stages of AMD may be a better strategy in sustaining visual function. This highlights the crucial role of timely detection and intervention in order to curb the advancement of the disease^8^.

Drusen are pathological indicators commonly associated with the early and intermediate stages of AMD^9^. In advanced AMD, drusen may diminish or be resorbed in areas of GA^10^. Growing evidence suggests that autophagy plays a role in the dysregulation of cellular waste clearance, contributing to drusen formation^11^. Notably, drusen deposits contain amyloid aggregates, including β-amyloid (Aβ), which is also a key pathological feature of Alzheimer’s disease (AD)^12^. Given that both AD and AMD are neurodegenerative disorders associated with aging, they may share common pathogenic mechanisms^13^.

**Fig. 1:**
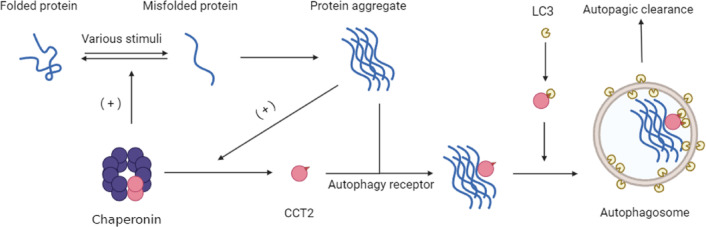
Mechanism of CCT2. The intricate mechanism of chaperonin containing TCP1 subunit 2 (CCT2) involves its active participation in the assembly of multifaceted CCT complexes with various subunits, functioning as steadfast molecular chaperones to ensure appropriate protein folding. The distinctive VLIR motif of CCT2 remains securely encapsulated within this complex structure. In response to an array of adverse stimuli, misfolded proteins cluster into aggregates, consequently initiating the detachment of CCT2 monomers from their incumbent molecular chaperones. These CCT2 monomers, in turn, tenaciously bind the protein aggregates through their apical domain. Intriguingly, the unshielded VLIR motif uniquely present on the monomeric CCT2 facilitates its binding to LC3, culminating in its function as a crucial autophagy receptor, thereby mediating an essential process known as aggregate autophagy.

Chaperonin containing TCP1 subunit 2 (CCT2), a molecular chaperone that plays a crucial role in protein homeostasis and various neurodegenerative diseases, is downregulated in AD and associated with the autophagic clearance of Aβ aggregates^14^. A recent study by Ma et al.^15^ demonstrated that molecular chaperones, represented by CCT2, can act as an aggregate autophagy receptor, aiding in aggregate clearance. CCT2, as a molecular chaperone, participates in the formation of the CCT complex with eight other subunits and plays a crucial role in protein folding. In this complex, the VLIR motif, which serves as the binding site of LC3 on CCT2, remains buried. CCT2 cannot act as an autophagy receptor in this state. However, the presence of aggregate proteins triggers the formation of CCT2 monomers. Monomeric CCT2 binds to protein aggregates via its apical domain, and the VLIR motif exposes on its surface, allowing CCT2 to interact with LC3 and function as an autophagy receptor, mediating aggregate autophagy (Fig. 1). This newly identified function of CCT2 suggests a potential role in AMD pathogenesis, particularly in drusen clearance, warranting further investigation.

Investigating the role of CCT2 in AMD may provide valuable insights into the disease’s pathogenesis and pave the way for novel therapeutic approaches. Given that the involvement of CCT2 in AMD pathogenesis remains unestablished, this study aims to examine changes in CCT2 expression levels in AMD patients and explore the potential autophagy-related pathways involved. By uncovering how CCT2 influences AMD through autophagy, our research may enhance the understanding of the disease’s underlying mechanisms and help inform future treatment strategies.

## Methods

### Data source and classification

This study was approved by the Ethical Committee of Shanxi Eye Hospital (NO. SXYYLL-20231003KS). The data for this study were obtained from the National Center for Biotechnology Information (NCBI) with the Gene Expression Omnibus (GEO) accession number GSE29801. The dataset comprises transcriptome profiles generated using expression profiling by array (microarray) from RPE-choroid and retina tissues of donor eyes. We specifically selected donor eyes categorized using the Rotterdam Grading Scale for AMD subtypes (Table 1^16,17^). To ensure consistency, samples from donors younger than 43 years of age were excluded. A total of 251 samples were included in the analysis, comprising 104 retina samples (53 macular and 51 extramacular) and 147 RPE samples (77 macular and 70 extramacular). To align with clinical practice and manifestations, we reclassified the samples according to the AMD classification established by the American Academy of Ophthalmology (AAO) (Table 2^18^). Specifically, MD1 and MD2 were classified as early AMD, dry AMD as intermediate AMD, and GA, CNV, and GA/CNV as advanced AMD. Gene expression values were log2-transformed for normalization to ensure comparability across samples.

**Table 1:** AMD classification scheme of GSE29801^16^.

AMD classification	Alternative name	Rotterdam grade ^17^	Rotterdam description	Donors per class*
MD1	Pre-AMD	0b	Hard macular drusen (< 63 μm) only	7
MD2	Sub-clinical pre-AMD	1a	Soft, distinct macular drusen (> 63 μm)	4 (1a only)
		1b	Macular pigmentary irregularities without soft drusen	
Dry AMD	Dry AMD (non-GA)	2a	Soft, indistinct (> 125 μm) or reticular macular drusen	17
		2b	Soft distinct macular drusen (> 63 μm) with pigmentary changes	17
		3	Soft indistinct macular drusen with pigmentary changes	17
GA	Geographic atrophy	4	Sharply demarcated area of apparent absence of the RPE (> 175 μm) involving central macular region	2
CNV	Wet AMD	4	Sub-retinal choroidal neovascularization	4
GA/CNV		4	Geographic atrophy with choroidal neovascularization	3

* Number of Iowa cohort donors per AMD classification group.

**Table 2:** AAO classification of AMD^18^.

Classification	Clinical manifestations
Early AMD	Defined by the presence of numerous small (< 63 microns, “hard”) or intermediate (≥ 63 microns but < 125 microns, “soft”) drusen
Intermediate AMD	Macular disease characterized by either extensive drusen of small or intermediate size, or any drusen of large size (≥ 125 microns)
Advanced AMD	Defined by the presence of either geographic atrophy or choroidal neovascular membrane (along with its sequelae, such as subretinal or sub-RPE hemorrhage or serous fluid, and subretinal fibrosis)

### CCT2 expression analysis and correlation with clinical features

We analyzed CCT2 expression in RPE-choroid and retina tissues from both macular and extramacular regions using R (version 4.3.0). The distribution of CCT2 expression across different AMD subtypes, age groups, genders, and regions were visualized using the R package ‘pheatmap’. When investigating the influence of age, samples were categorized into four age groups: ≤70 years, 70–80 (inclusive) years, 80–90 (inclusive) years, and > 90 years. Differential expression analysis was performed using the ‘limma’ package, which employs a linear model to assess statistical significance. The distribution of CCT2 expression levels across groups was visualized using boxplots generated with the ggboxplot function.

### Receiver operating characteristic (ROC) curve analysis

To evaluate the diagnostic potential of CCT2 in advanced AMD stage, AMD samples were divided into two groups: non-advanced (early and intermediate AMD) and advanced AMD. ROC curves were generated for macular and extramacular retina using the R package ‘pROC’. The area under the curve (AUC) was calculated to assess the discriminatory power of CCT2 expression levels.

### Pathway analysis of CCT2

Pathway analysis related to CCT2 was conducted using the Reactome database. We utilized the “Analysis Tools” feature, entered “CCT2” in the search box, and selected “project to human” as the preferred option. The most relevant pathways associated with CCT2 were identified and downloaded for further analysis.

### Functional enrichment analysis

The correlation between CCT2 expression and other genes was assessed using the cor.test function from the ‘limma’ package. Genes with absolute Pearson correlation coefficient |R| > 0.5 and *p* < 0.001 were considered significantly correlated (Supplementary Tables 1–2). In the macular retina, 386 genes (272 positively and 114 negatively correlated) showed significant correlations with CCT2 (Supplementary Table 1), while 1,250 genes (950 positively and 301 negatively correlated) were identified in the extramacular retina (Supplementary Table 2). These positively correlated genes were then subjected to functional enrichment analysis using the Database for Annotation, Visualization, and Integrated Discovery (DAVID, v6.8)^19–21^. Official gene symbols were used as identifiers, and Homo sapiens was chosen as the species. Gene Ontology (GO) terms and Kyoto Encyclopedia of Genes and Genomes (KEGG) pathways analyses were performed. The top six GO terms and top ten KEGG pathways, ranked by ascending p-values, were reported.

### Single sample gene set enrichment analysis (ssGSEA)

Samples were divided into two groups, non-advanced (early and intermediate AMD) and advanced stage. Gene sets related to autophagy and protein folding were selected from the C2 (curated gene sets) and C5 (GO gene sets: Gene Ontology) categories, downloaded from the Molecular Signatures Database (MSigDB). Gene Set Variation Analysis (GSVA) analysis was conducted using the ‘GSVA’ package with default parameters. Heatmaps showing the enrichment scores of 12 selected gene sets in both macular and extramacular retina were generated using the ‘pheatmap’ package. The Pearson correlation coefficients between CCT2 expression levels and these gene sets, along with their corresponding p-values, were displayed in bar charts.

### CCT2 and autophagy-related genes

A set of 222 autophagy-related genes was obtained from the Human Autophagy Database (HADb). To explore the relationship between CCT2 and autophagy genes, we identified the intersection between CCT2-related genes in the retina and the autophagy gene set. In the macular retina, all 386 CCT2-correlated genes were selected, and for comparability, an equal number of CCT2-related genes from the extramacular retina was chosen, ranked by |R|. The interactions between CCT2 and these intersecting genes were visualized using circle diagrams generated with the ‘circlize’ package.

### Relationship between CCT2 and AMD etiology

A correlation matrix was constructed to evaluate the relationship between CCT2 expression levels and established AMD-related factors^22,23^, including hypoxia, oxidative stress, autophagy formation, macroautophagy, molecular chaperone-mediated autophagy, mTOR pathway, and epithelial-mesenchymal transition (EMT). Gene sets associated with these mechanisms were retrieved from the C2 (curated gene sets) and C5 (GO gene sets: Gene Ontology) categories in the MSigDB. GSVA was performed using the gsva function to calculate enrichment scores for each sample, retaining gene sets with at least one overlapping gene. The correlation matrix was visualized using the ‘corrgram’ package to depict relationships between them.

### Cell culture and sodium iodate treatment

661 W cells were cultured in Dulbecco’s Modified Eagle Medium (DMEM) supplemented with 10% fetal bovine serum (FBS) and 1% penicillin-streptomycin at 37 °C in a humidified atmosphere containing 5% CO_2_. To simulate AMD conditions, 661 W cells were treated with sodium iodate (NaIO_3,_ MCE, HY-Y0628) at a final concentration of 15 mM for 24 h.

### RNA extraction and quantitative real-time PCR (qRT-PCR)

Total RNA was extracted from 661 W cells using the FastPure RNA Kit according to the manufacturer’s protocol. Briefly, cells were lysed in Buffer RL, and the lysate was processed through FastPure gDNA-Filter and RNA Columns with sequential washes using Buffer RW1 and RW2. RNA was eluted in RNase-free water and stored at -80°C. The purity and concentration of RNA were assessed using a NanoDrop spectrophotometer, with an OD260/OD280 ratio > 1.8 indicating sufficient purity. cDNA was synthesized using the ABScript III RT Kit with gDNA removal at 37°C for 2 min, followed by reverse transcription at 55°C for 15 min and enzyme inactivation at 85°C for 5 min. qRT-PCR was performed using Genious 2X SYBR Green Fast RT-qPCR Mix on a real-time PCR system under the following cycling conditions: initial denaturation at 95°C for 3 min, followed by 40–45 cycles of 95°C for 5 s and 60°C for 30 s. β-actin served as the internal control, and the primers for CCT2 were as follows: forward 5’-TGACCAACGACGGTGCTACCAT-3’ and reverse 5’-ACAGAGGTAGTGCCATCACCAAC-3’. Primers for β-actin: forward 5’-CATTGCTGACAGGATGCAGAAGG-3’ and reverse 5’-TGCTGGAAGGTGGACAGTGAGG-3’. The relative expression levels of CCT2 were determined using the 2^(-ΔΔCt) method. Melt curve analysis was conducted to verify amplification specificity.

### Statistical analysis

Normality of data distribution was assessed using the Shapiro-Wilk test, and homogeneity of variance was evaluated using Levene’s test. For data with normal distribution and equal variance, comparisons between two groups were performed using the Student’s t-test, while one-way ANOVA followed by Tukey’s post hoc test was used for multiple group comparisons. For non-normally distributed data or data with unequal variance, the Wilcoxon rank-sum test was used for two-group comparisons, and the Kruskal-Wallis test was applied for multiple group comparisons. All Pearson correlation tests were two-tailed.

The qRT-PCR experiments were performed in triplicate. Data were presented as mean ± standard deviation (SD). Statistical significance was assessed using a two-tailed Student’s t-test in R. Statistical significance was set at *p* < 0.05.

## Results

### Expression level of CCT2 with AMD clinical features

**Fig. 2:**
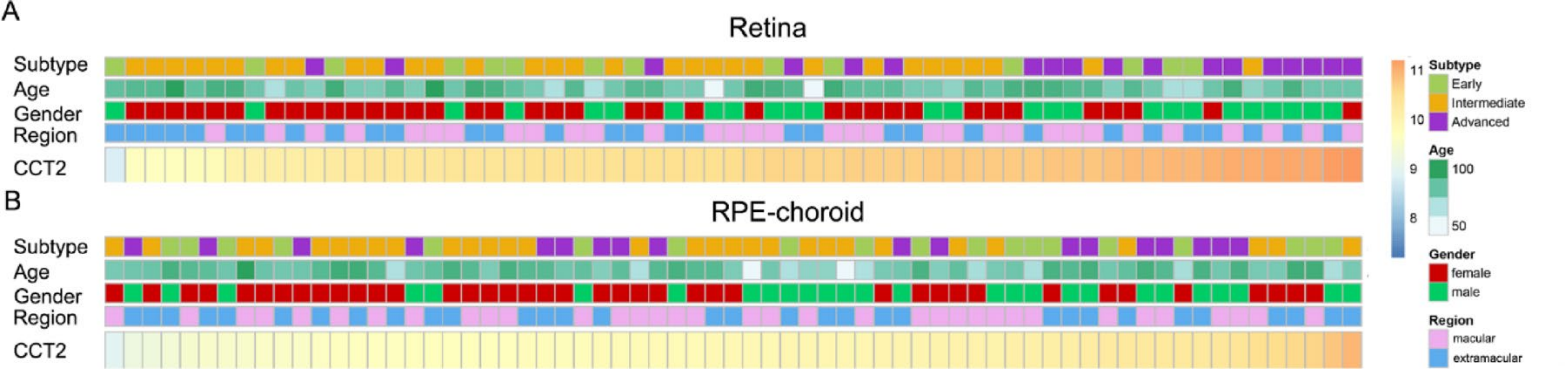
Embarking on a meticulous construction of a comprehensive distribution of the diverse clinical and pathological features in age-related macular degeneration (AMD). (**A**) A detailed describe of the distribution of varying chaperonin containing TCP1 subunit 2 (CCT2) expression levels, distinct AMD subtypes, a broad age spectrum, gender, and the specific region in the retina. (**B**) The systematic survey of the distribution of different CCT2 expression levels, specific AMD subtype, encompassing all age groups, gender, and region within the RPE-choroid.

**Fig. 3:**
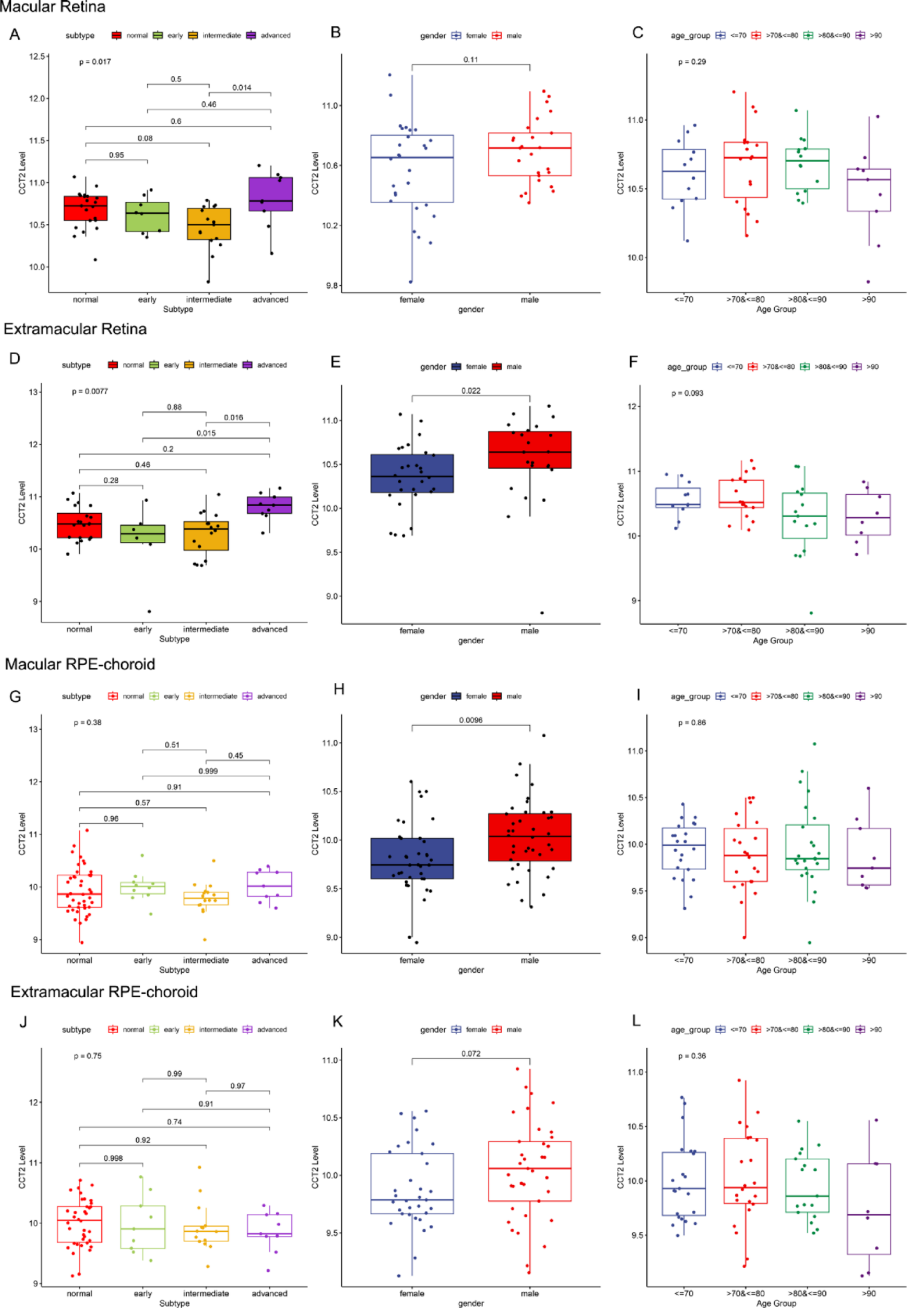
Expression levels of CCT2 across AMD subtypes, gender, and age groups. (**A**–**C**) Macular retina: (**A**) AMD subtype, (**B**) gender, (**C**) age group. (**D**–**F**) Extramacular retina: (**D**) AMD subtype, (**E**) gender, (**F**) age group. (**G**–**I**) Macular RPE-choroid: (**G**) AMD subtype, (**H**) gender, (**I**) age group. (**J**–**L**) Extramacular RPE-choroid: (**J**) AMD subtype, (**K**) gender, (**L**) age group.

Patients with varying levels of CCT2 expression showed clear differences in clinical and pathological characteristics. Upon analyzing the RPE-choroid and retina datasets, an uneven distribution of CCT2 expression was observed based on AMD subtype, age, gender, and source region (Fig. 2). However, prominent statistically significant differences in CCT2 expression were observed only across subtypes and between genders, and no age-related variations were detected in any samples (Fig. 3). Specifically, in the retinal layer, CCT2 expression was significantly higher in the macular region of advanced AMD compared to intermediate AMD (Fig. 3A). Similarly, in the extramacular retina, advanced AMD exhibited higher CCT2 levels than both early and intermediate AMD (Fig. 3D). Moreover, in the extramacular retina and macular RPE, males exhibited significantly higher CCT2 expression than females (Fig. 3E, H). Within the retinal layer, CCT2 expression was higher in the macular region than in the extramacular region, whereas no significant differences were found between different regions in the RPE layer (Supplementary Fig. 1). Given these findings, only retinal layer data were included in subsequent analyses.

### CCT2 as a potential biomarker for advanced AMD

**Fig. 4:**
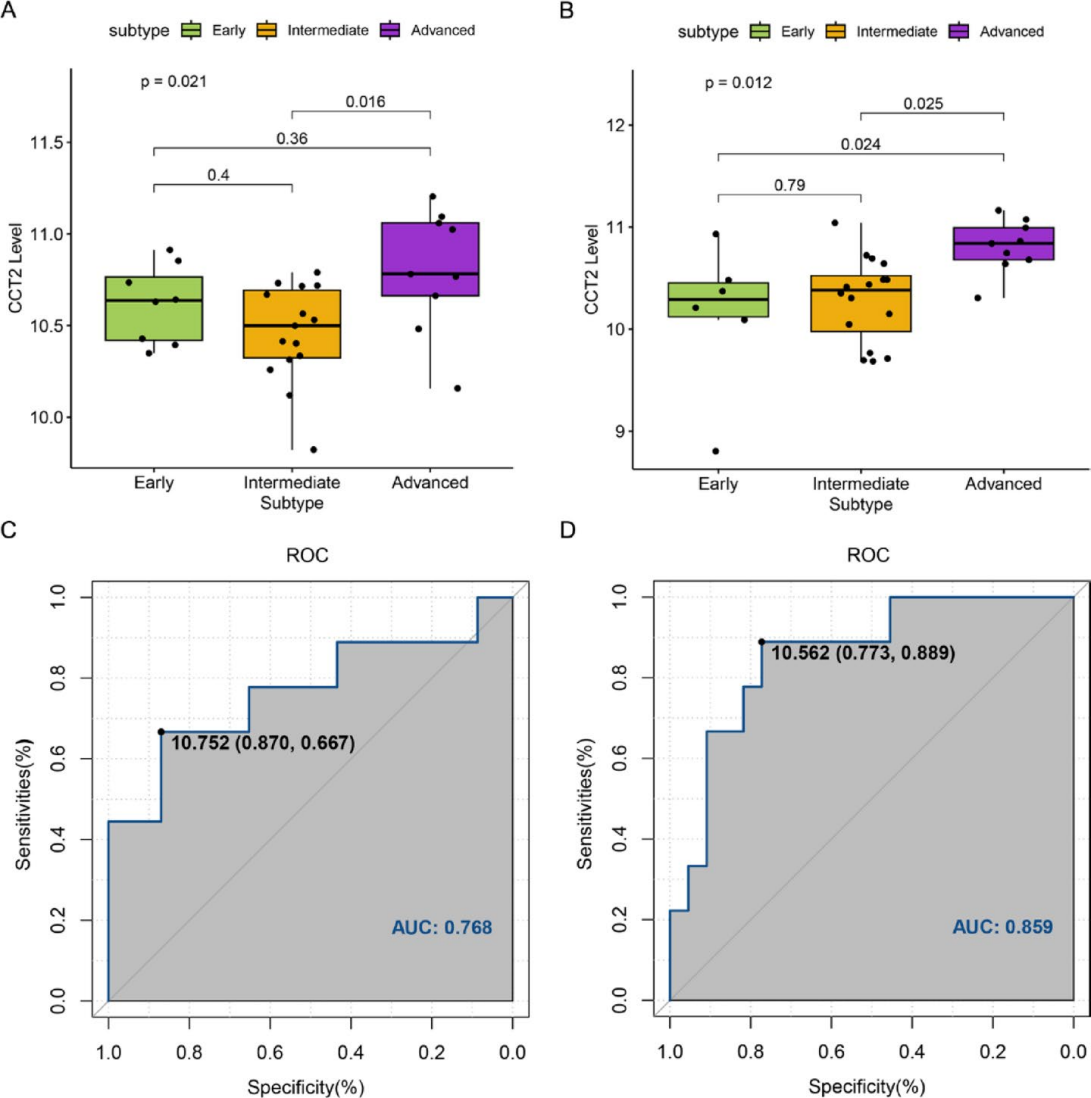
Unveiling the comprehensive distribution of chaperonin containing TCP1 subunit 2 (CCT2) across various age-related macular degeneration (AMD) subtypes and diagnostic significance in advanced AMD. (**A**) Detailed investigation into the expression of CCT2 across diverse American Academy of Ophthalmology (AAO) AMD subtypes, as derived from an extensive macular retina database. (**B**) Delving more deeply into the expression of CCT2 in distinct AAO AMD subtypes, extracted from a far-reaching extramacular retina database. (**C**) An accurate receiver operating characteristic (ROC) curve displays the specificity of CCT2 expression in the context of advanced AMD (macular retina). (**D**) Similarly, a precision ROC curve illustrates the specificity of CCT2 expression in advanced AMD (extramacular retina).

CCT2 was significantly enriched in advanced AMD compared to intermediate AMD (*p* < 0.05) (Fig. 4A, B). The diagnostic performance of CCT2 for distinguishing advanced AMD from non-advanced stage yielded an area under the curve (AUC) of 76.8% in the macular retina (Fig. 4C) and 85.9% in the extramacular retina (Fig. 4D). However, substantial variability was observed among patients, indicating that while CCT2 may serve as a potential biomarker, further validation is required.

### Functional overview of CCT2 (Reactome pathway analysis)

A genome-wide Reactome pathway analysis revealed that CCT2 is involved in protein metabolism, organelle biogenesis and maintenance, signal transduction, and the immune system (Supplementary Fig. 2). The 23 most relevant pathways, ranked by p-value, suggest that CCT2 primarily participates in protein folding process (Table 3). Misfolded proteins play a pivotal role in promoting the formation of CCT2 monomers, which subsequently interact with misfolded proteins and may contribute to aggregate clearance through autophagy^15^. Given the established link between autophagy and drusen turnover, the elevated expression of CCT2 in advanced AMD may be associated with enhanced autophagy-mediated clearance of drusen deposits. This suggests that CCT2 upregulation could represent an adaptive cellular response to mitigate extracellular debris accumulation. However, further functional studies are necessary to confirm this hypothesis and elucidate the precise role of CCT2 in AMD pathophysiology.

**Table 3:** The 23 most relevant pathways of CCT2 sorted by p-value.

Pathway name	Entities				Reactions	
	Found	Ratio	*p*-value	FDR*	Found	Ratio
Folding of actin by CCT/TriC	1/10	8.62E−04	8.62E−04	6.04E−03	2/2	1.45E−04
RHOBTB1 GTPase cycle	1/23	1.98E−03	1.98E−03	6.04E−03	1/2	1.45E−04
RHOBTB2 GTPase cycle	1/23	1.98E−03	1.98E−03	6.04E−03	1/2	1.45E−04
BBSomE−mediated cargo-targeting to cilium	1/23	1.98E−03	1.98E−03	6.04E−03	1/5	3.62E−04
Formation of tubulin folding intermediates by CCT/TriC	1/26	2.24E−03	2.24E−03	6.04E−03	2/2	1.45E−04
Prefoldin mediated transfer of substrate to CCT/TriC	1/28	2.41E−03	2.41E−03	6.04E−03	1/2	1.45E−04
Cooperation of Prefoldin and TriC/CCT in actin and tubulin folding	1/33	2.85E−03	2.85E−03	6.04E−03	5/6	4.34E−04
RHOBTB GTPase Cycle	1/35	3.02E−03	3.02E−03	6.04E−03	2/4	2.89E−04
Cooperation of PDCL (PhLP1) and TRiC/CCT in G-protein beta folding	1/38	3.28E−03	3.28E−03	6.55E−03	8/11	7.95E−04
Association of TriC/CCT with target proteins during biosynthesis	1/39	3.36E−03	3.36E−03	6.73E−03	2/2	1.45E−04
Cargo trafficking to the periciliary membrane	1/51	4.40E−03	4.40E−03	7.93E−03	1/27	1.95E−03
Chaperonin-mediated protein folding	1/92	7.93E−03	7.93E−03	7.93E−03	15/19	1.37E−03
Protein folding	1/98	8.45E−03	8.45E−03	8.45E−03	15/28	2.02E−03
Cilium Assembly	1/202	1.74E−02	1.74E−02	1.74E−02	1/50	3.62E−03
Organelle biogenesis and maintenance	1/298	2.57E−02	2.57E−02	2.57E−02	1/86	6.22E−03
RHO GTPase cycle	1/452	3.90E−02	3.90E−02	3.90E−02	2/91	6.58E−03
Neutrophil degranulation	1/478	4.12E−02	4.12E−02	4.12E−02	1/10	7.23E−04
Signaling by Rho GTPases	1/677	5.84E−02	5.84E−02	5.84E−02	2/203	1.47E−02
Signaling by Rho GTPases, Miro GTPases and RHOBTB3	1/693	5.98E−02	5.98E−02	5.98E−02	2/212	1.53E−02
Innate Immune System	1/1196	1.03E−01	1.03E−01	1.03E−01	1/690	4.99E−02
Metabolism of proteins	1/1949	1.68E−01	1.68E−01	1.68E−01	15/795	5.75E−02
Immune System	1/2186	1.89E−01	1.89E−01	1.89E−01	1/1623	1.17E−01
Signal Transduction	1/2598	2.24E−01	2.24E−01	2.24E−01	2/2530	1.83E−01

1 out of 1 identifiers in the sample were found in Reactome, where 23 pathways were hit by at least one of them.

*False Discovery Rate.

### Association between CCT2 and autophagy pathways

**Fig. 5:**
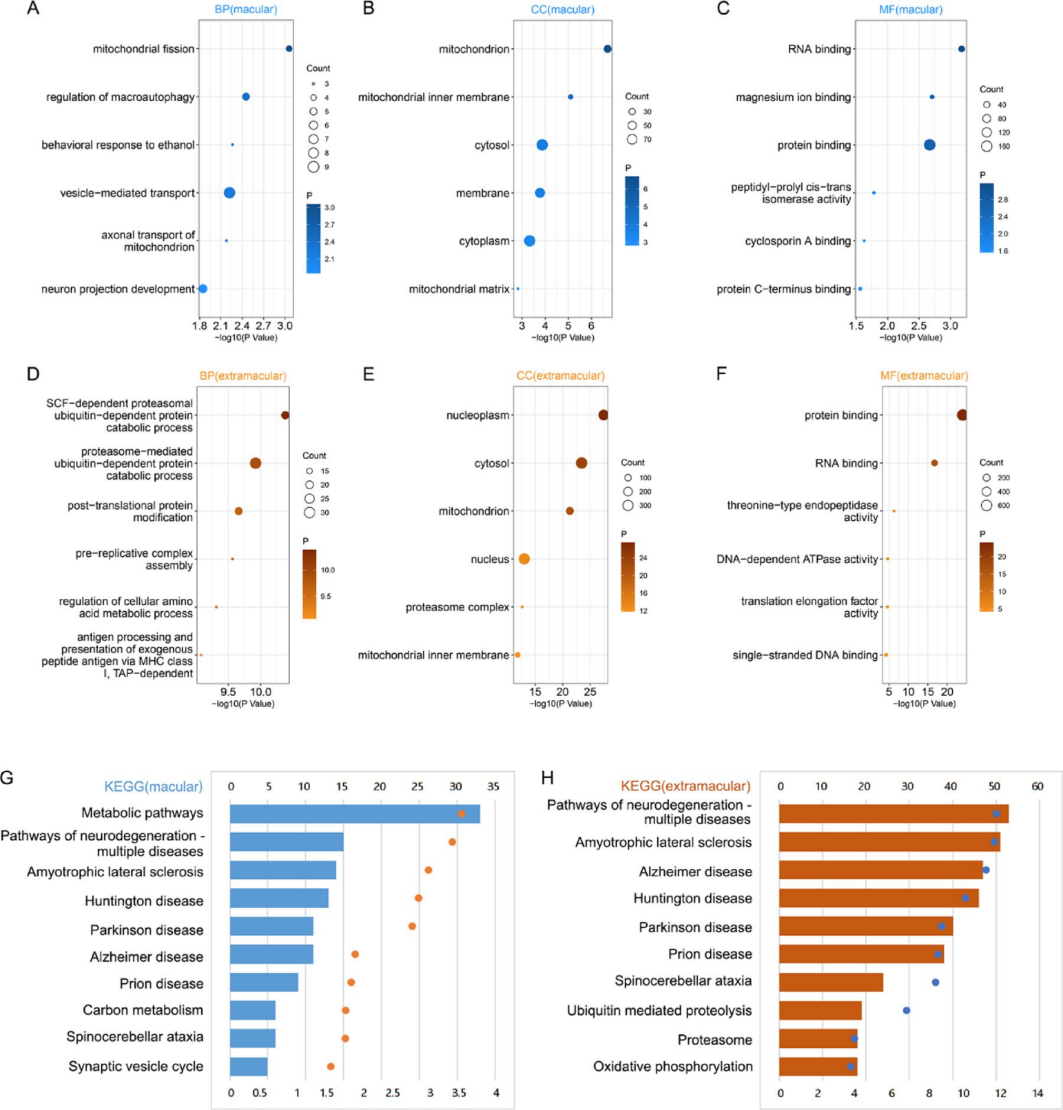
Devise an exhaustive terms enrichment, focusing on chaperonin containing TCP1 subunit 2 (CCT2)-positively related genes within the retina. This includes a meticulously enriched Biological Process (BP) in the fascinating macular region (**A**), along with enriched Cellular Component (CC) (**B**), and Molecular Function (MF) (**C**). Likewise, in the intriguing extramacular region, there is an enriched BP (**D**), CC (**E**), and MF (**F**). Notably, the confidence progressively intensifies from light to dark color, with the circle sizes indicating the number of gene constituents within the corresponding pathway. The analysis also boasts enriched Kyoto Encyclopedia of Genes and Genomes (KEGG) pathways^19–21^ in both the macular region (G) and the extramacular regions (H). The coordinates located below each circle mirror the confidence level, while those placed above the bars represent the volume of genes incorporated within the respective pathway.

There were 272 genes positively correlated with CCT2 in the macular retina (Supplementary Table 1) and 950 genes in the extramacular retina (Supplementary Table 2) through Pearson correlation analysis (*R* > 0.5, *p* < 0.001). In the macular retina, CCT2 was associated with biological processes (BP) such as mitochondrial fission, regulation of macroautophagy, behavioral response to ethanol, vesicle-mediated transport, axonal transport of mitochondrion, and neuron projection development (Fig. 5A). The associated cellular components (CC) included mitochondrion, mitochondrial inner membrane, cytosol, membrane, cytoplasm, and mitochondrial matrix (Fig. 5B), while molecular functions (MF) encompassed RNA binding, magnesium ion binding, protein binding, peptidyl-prolyl cis-trans isomerase activity, cyclosporin A binding, and protein C-terminus binding (Fig. 5C). Pathway analysis revealed that the top 10 pathways related to CCT2 were primarily linked to various neurodegeneration diseases and metabolism, including metabolic pathways, pathways of neurodegeneration-multiple diseases, carbon metabolism and so on (Fig. 5G).

These findings suggest that CCT2 likely regulates diverse biological processes, particularly in retinal function and neurodegeneration. It is involved in mitochondrial dynamics, autophagy, neuronal development, and protein folding, with additional roles in RNA interactions and ion binding. Furthermore, CCT2 may contribute to neurodegenerative diseases and metabolic dysregulation through its involvement in mitochondrial function and protein homeostasis, highlighting its potential importance in retinal health and disease. Notably, similar results were observed in the extramacular dataset, indicating a conserved role for CCT2 across different retinal regions (Fig. 5D–F, H).

### Enrichment analysis of CCT2 and autophagy-related processes

**Fig. 6:**
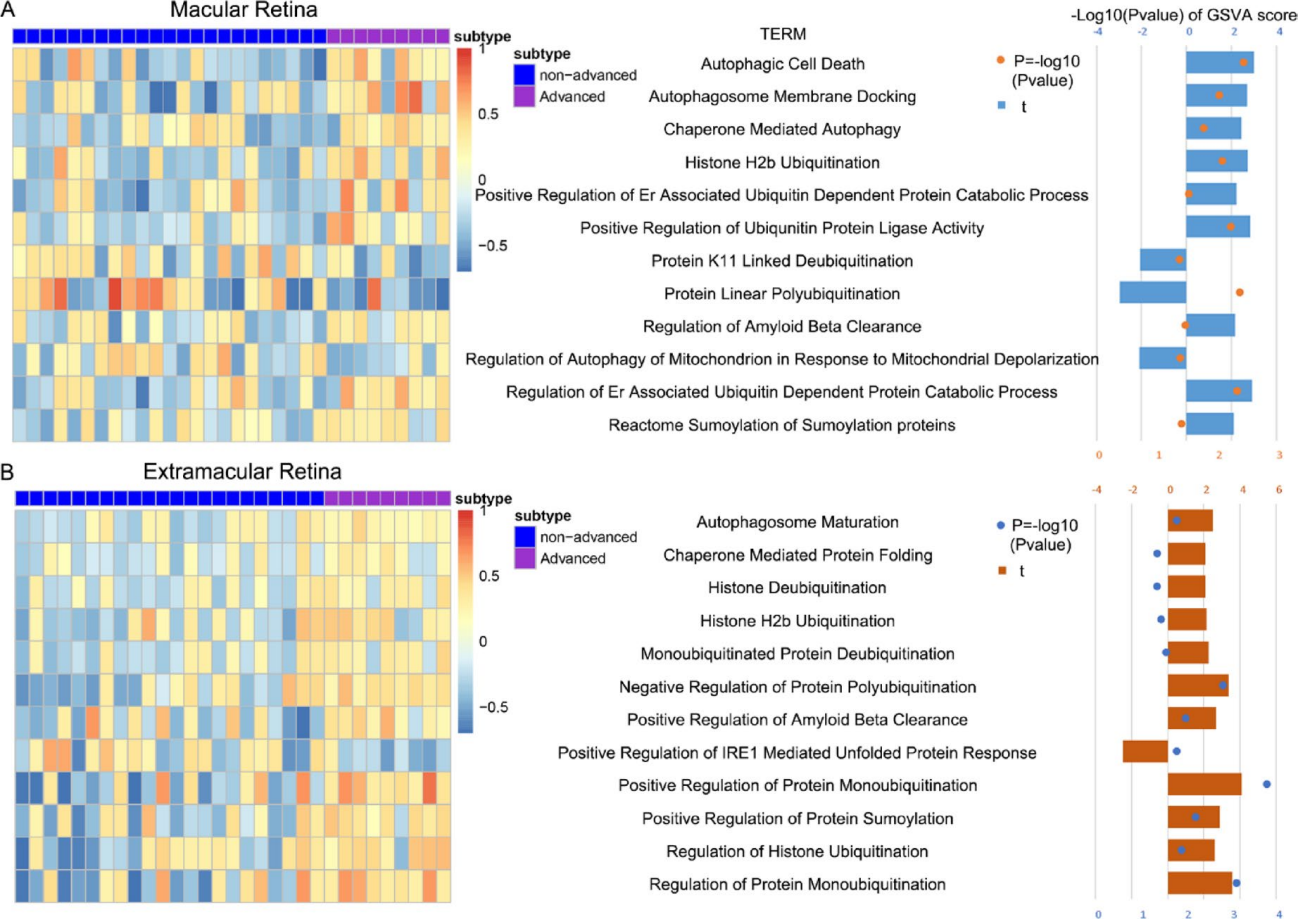
Enrichment analysis using ssGSEA. Conjure an illuminating and instructive heatmap on the left that vividly exhibits the enrichment scores of age-related macular degeneration (AMD) subtypes and the interconnected pathways in the comprehensive GSE29801 dataset. The meticulously chosen samples for this groundbreaking study were neatly divided into two distinct groups: the non-advanced group and the advanced group. Within each group, the expression of the pivotal chaperonin containing TCP1 subunit 2 (CCT2) was methodically arranged in ascending order, with the corresponding, relevant gene set names strategically placed in the heart of the layout. The critical t and p values for the insightful differential analysis are conspicuously displayed in the right bar. This study was meticulously conducted in two specific regions: (**A**) the central macular region, and (**B**) the peripheral extramacular region.

Given CCT2’s enrichment in advanced AMD, we divided AMD cases into non-advanced group with low CCT2 level and advanced group with high CCT2 level. Enrichment analysis using ssGSEA indicated that CCT2 was positively correlated with several autophagy-related processes. In the macular dataset, CCT2 was positively correlated with various autophagy functions, except for specific processes like protein K11 linked deubiquitination, protein linear polyubiquitination, and regulation of autophagy of mitochondrion in response to mitochondrial depolarization (Fig. 6A). In the extramacular dataset, only positive regulation of Ire1-mediated unfolded protein response exhibited a negative correlation with CCT2 levels (Fig. 6B). These findings suggest that CCT2 may be involved in autophagy regulation.

### Relationship between CCT2, autophagy-related genes and AMD pathogenesis

**Fig. 7:**
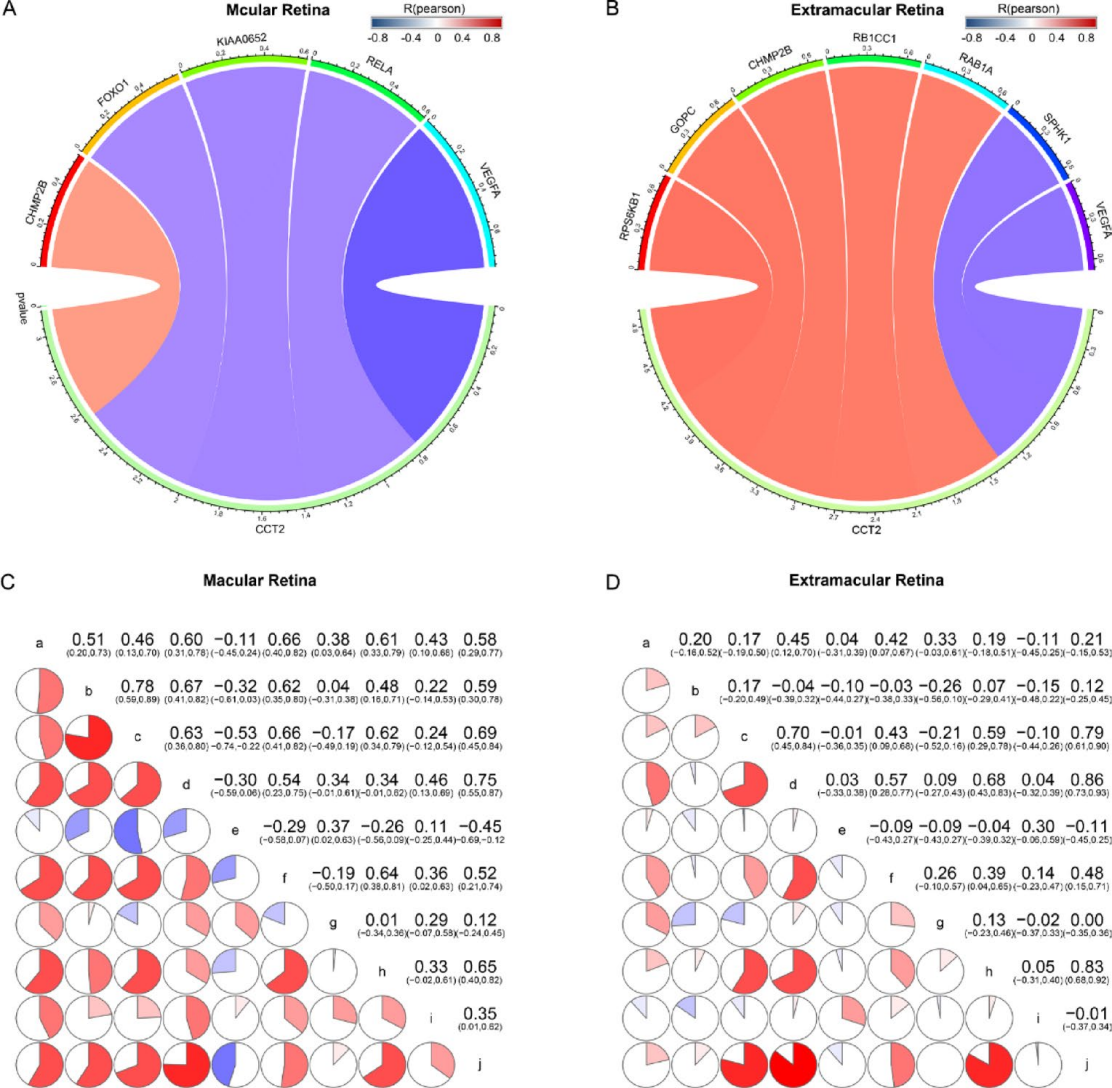
Craft a meticulously comprehensive association of chaperonin containing TCP1 subunit 2 (CCT2) with autophagy-related genes and some rigorously validated pathogenesis of age-related macular degeneration (AMD). (**A**) Correlation between CCT2 and autophagy-related genes in the macular region. (**B**) Correlation between CCT2 and autophagy-related genes in the extramacular region. (**C**) Association of CCT2 with some validated AMD pathogenesis in the macular region. (**D**) Association of CCT2 with some validated AMD pathogenesis in the extramacular region. a: neuroinflammation (WP), b: neuroinflammation (HP), c: aging (GOBP), d: EMT down (SARRIO), e: EMT up (SARRIO), f: oxidative stress response (WP), g: oxidative stress induced senescence (REACTOME), h: response to oxidative stress (GOBP), i: neuron death in response to oxidative stress (GOBP), j: response to reactive oxygen species (GOBP).

CCT2 has been linked to aggregate clearance through autophagy, prompting an analysis of its relationship with autophagy-related genes. We identified overlapping genes between CCT2-related genes and autophagy gene sets, comprising 5 genes in the macular region and 7 genes in the extramacular region (Fig. 7A, B). As shown in Fig. 7A, CCT2 displayed positive correlations with CHMP2B and negative correlations with FOXO1, KIAA0652, RELA, and VEGFA in the macular retina. In the extramacular retina, CCT2 showed positive correlations with RPS6KB1, GOPC, CHMP2B, RB1CC1, and RAB1A, and negative correlations with SPHK1 and VEGFA (Fig. 7B). These associations suggest a potential role for CCT2 in autophagy regulation; however, the directionality and functional significance of these correlations require further investigation.

To explore CCT2’s association with AMD-related biological processes, we examined correlations with ten gene sets associated with known AMD pathogenesis, including neuroinflammation, aging, EMT, oxidative stress response, oxidative stress-induced senescence, neuron death in response to oxidative stress, and response to reactive oxygen species. Correlation matrix diagrams demonstrated possible associations between CCT2 expression and the likely pathogenesis of AMD (Fig. 7C, D).

### Quantitative real-time PCR (RT-qPCR)

**Fig. 8:**
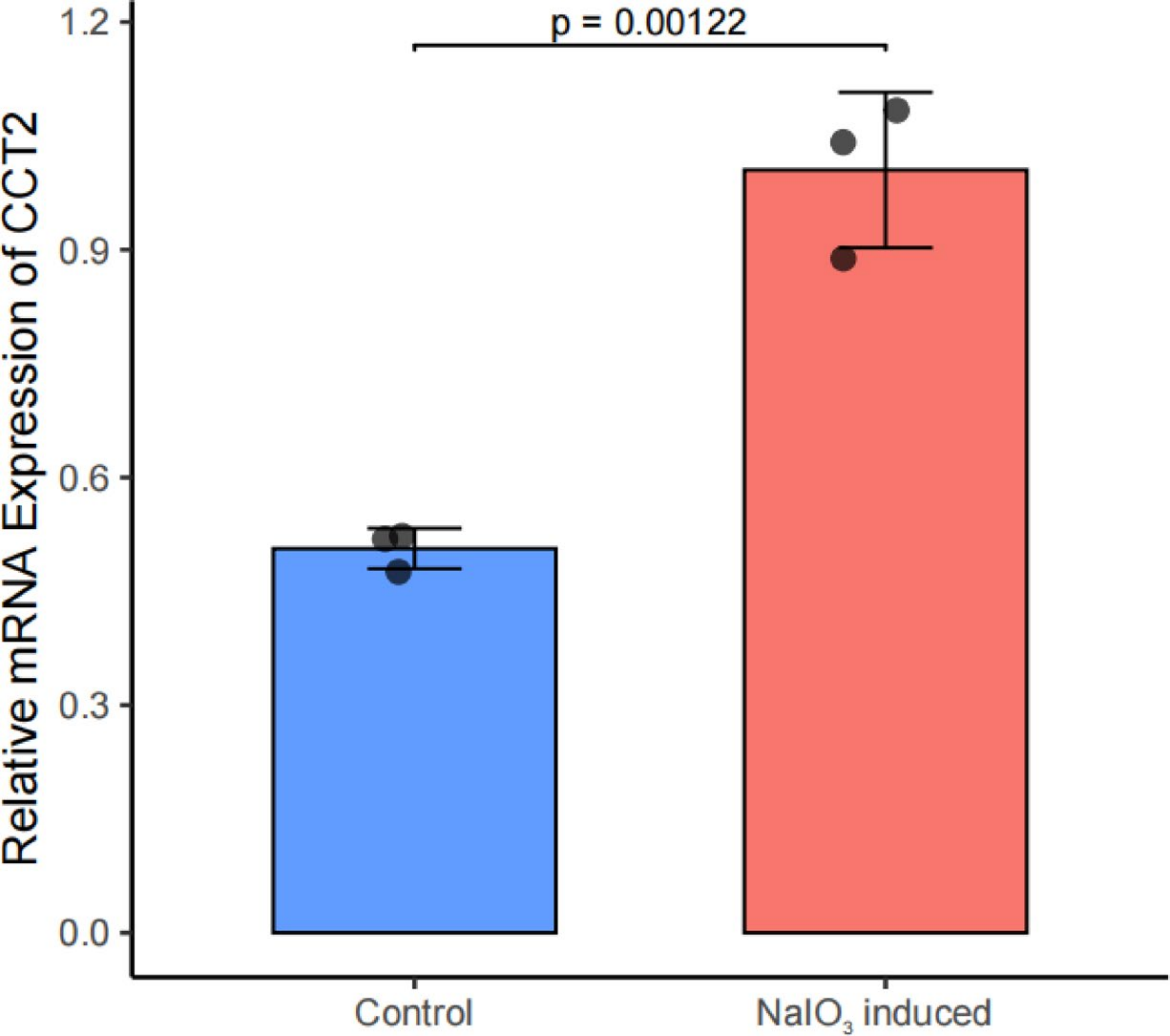
The expression level of CCT2 in 661 W cells treated with sodium iodate assessed by qPCR. *p* < 0.01.

To further validate the expression level of CCT2, we treated 661 W cells with NaIO_3_ to model AMD. RT-qPCR revealed a significant upregulation of CCT2 expression following NaIO_3_ treatment (*p* < 0.01) (Fig. 8). This trend aligns with our transcriptomic data, further supporting the role of CCT2 in advanced AMD progression.

## Discussion

Autophagy, a conserved cellular process in eukaryotes, involves the degradation of damaged organelles and misfolded proteins via the lysosomal pathway, recycling small molecules and amino acids to maintain cellular homeostasis^24^. Autophagy helps maintain retinal homeostasis, and its dysfunction can lead to retinal cell death and accelerate AMD pathology^11^. Its involvement in AMD progression is further supported by altered serum levels of autophagy-related proteins like Beclin-1 and mTOR in exudative AMD patients^25^. Additionally, prior studies have linked autophagy to Aβ production and metabolism, highlighting its broader role in neurodegenerative processes^26^.

CCT2, a molecular chaperone, is essential for maintaining protein homeostasis by regulating protein folding, degradation, and autophagy. Beyond its chaperone function, CCT2 also acts as a novel ubiquitin-binding receptor for aggregate autophagy, contributing to the clearance of various toxic protein aggregates associated with neurodegenerative diseases^27^. This will provide a better consideration for targeting CCT2 for the treatment of aggregate related diseases^15^. These known roles of CCT2 in protein homeostasis suggest it may be relevant to the formation or regression of drusen, though its specific role in AMD context remains to be clarified.

In this study, we observed that CCT2 expression was significantly upregulated in the retina of advanced AMD compared to intermediate AMD, suggesting a potential role in disease progression. This finding was further validated in an in vitro model using NaIO_3_-induced 661 W cells (photoreceptor cells of mouse retina). While CCT2 expression varied significantly between intermediate and advanced AMD stages, no significant changes were observed between any stage with normal samples, indicating that CCT2 upregulation may be more relevant to advanced-stage disease processes rather than serving as an early biomarker. Additionally, inter-individual variability in CCT2 expression was evident, underscoring the need for larger cohort studies to confirm these findings. MITTER SK et al. demonstrated that acute oxidative stress enhances autophagic activity in RPE cells, while chronic oxidative stress leads to autophagy suppression. Their studies further revealed a biphasic pattern of autophagy dysregulation during AMD progression, characterized by enhanced autophagic clearance in early AMD stages and significant impairment in advanced disease, as evidenced by reduced levels of autophagic proteins, autophagosomes, and autophagic flux in both human donor AMD eyes and mouse models^28^. Our data revealed a stage-specific pattern of CCT2 expression that appears to aligns with this biphasic model, although we recognize that this correlation does not imply causation. Specifically, we observed a trend toward decreased CCT2 expression in early and intermediate AMD stages (albeit not statistically significant), and an upregulation in advanced AMD. This association may reflect changes in autophagy activity across disease stages, but further mechanistic studies are needed to confirm any functional involvement. Therefore, any potential implications for autophagy-targeting therapies must be interpreted cautiously. The observed increase in CCT2 expression in advanced AMD may coincides with the limited efficacy of autophagy-targeting therapeutic strategies (such as mTOR inhibition, a major negative regulator of autophagy initiation) in clinical trials for advanced AMD^29,30^. This paradox may stem from the late recruitment of patients into the AMD study. Our findings suggest that the timing of autophagy modulation may be critical, as autophagy showed different tendency in AMD stages.

While our transcriptomic analysis revealed significant differential expression of CCT2 in the retina but not in the RPE/choroid complex, we believe this does not preclude its potential involvement in autophagy regulation and drusen clearance for several reasons. First, previous studies have highlighted the role of retina-derived signaling molecules in modulating RPE function^31^. It is therefore possible that retinal CCT2 expression changes reflect or contribute to altered intercellular communication that secondarily influences autophagic activity in the RPE. Second, photoreceptors and Müller glia are now recognized as key players in AMD pathophysiology and homeostatic regulation, alongside RPE cells^32,33^. Given that photoreceptors are highly metabolically active and rely on tight metabolic coupling with the RPE, perturbations in retinal protein quality control pathways, including chaperone-mediated autophagy (to which CCT2 contributes), may indirectly affect RPE clearance capacity. Third, the absence of significant differential expression in RPE/choroid samples might be due to tissue heterogeneity, variability in donor stages, or lower RNA abundance of CCT2 in these layers compared to retina, potentially affecting detection sensitivity. Taken together, although CCT2 expression changes were only observed in the retina, they may still reflect or contribute to broader disruptions in proteostasis and autophagy during AMD progression. Nonetheless, these interpretations are preliminary and further studies using spatial transcriptomics or single-cell RNA sequencing will be instrumental in delineating these compartment-specific roles.

Notably, we also observed higher CCT2 expression levels in males compared to females in some samples. This aligns with previous studies suggesting sex-based differences in AMD susceptibility and progression. Rudnicka et al. conducted a meta-analysis of European populations and reported that females are more susceptible to advanced stages of the disease^34^. This disparity may be attributed to differences in hormonal regulation, genetic factors, or epigenetic modifications between sexes. Our findings suggest a potential association between CCT2 expression and the observed sex-based disparity in AMD susceptibility, though the exact mechanistic link remains unclear.

The functional enrichment analysis of genes positively correlated with CCT2 highlighted their role in maintaining protein homeostasis. Specifically, these genes are involved in regulating macroautophagy, protein binding, the ubiquitin-dependent protein catabolic process, and post-translational protein modifications. These findings align with the proposed role of CCT2 in regulating protein homeostasis through molecular chaperone activity and aggregate autophagy^15^. Meanwhile, pathway enrichment analysis showed that CCT2 is associated with multiple neurodegenerative diseases in animals, which is consistent with previous findings^14,35^.

Our GSVA analysis of CCT2-related genes in retina from AMD patients showed that CCT2 is closely related to various biological processes such as initiation of autophagy, chaperon-mediated autophagy, mitochondrial autophagy, protein ubiquitination and deubiquitination, protein folding and catabolism. In both macular and extramacular regions, CCT2 was positively correlated with most of the autophagy related gene sets. It also suggests that CCT2 may be involved in the occurrence and progression of AMD by affecting the occurrence of autophagy through some mechanism. In addition, CCT2 may also affect AMD through autophagic cell death and Aβ clearance regulation.

When considering the link between CCT2 and autophagy-related genes, CCT2 was positively correlated with CHMP2B, and negatively correlated with VEGFA in both macular and extramacular retina. The research on CHMP2B is mainly focused on autophagy, frontotemporal dementia and amyotrophic lateral sclerosis^36–38^, suggesting that CHMP2B could be one of the targets through which CCT2 participated in autophagy and affecting the course of AMD. VEGFA is well known as an important pathogenesis and therapeutic target of wet AMD. Future studies about CCT2 and VEGFA may link CCT2 with neovascularization. In order to further explore the specific mechanism of CCT2 involvement in AMD, we analyzed its relationship with various established causes of AMD. The results suggest that CCT2 can influence AMD in multiple ways, either promoting or inhibiting it, and may be linked to almost every aspect of the disease. However, it is important to note that our study is based on transcriptomic analysis, which does not establish causality. Further functional studies, such as knockdown or overexpression experiments, are necessary to elucidate the precise mechanisms by which CCT2 influences AMD pathogenesis, which goes beyond the scope of our current research.

## Conclusions

Our study highlights the upregulation of CCT2 in advanced AMD, suggesting its role in late-stage disease progression through autophagy and protein homeostasis regulation. Sex-based differences in CCT2 levels were observed, with higher expression in males, potentially indicating a protective mechanism. Functional analyses linked CCT2 to key autophagy-related processes and neurodegenerative pathways, supporting its involvement in AMD pathogenesis. Notably, CCT2 showed correlations with genes such as CHMP2B and VEGFA, suggesting potential roles in autophagy regulation and neovascularization. However, our study is limited by its reliance on transcriptomic data, which precludes causal inferences. Further functional studies are needed to confirm these findings and explore CCT2 as a potential therapeutic target.

## Electronic supplementary material

Below is the link to the electronic supplementary material.

Supplementary Material 1

## Data Availability

All data generated or analysed during this study are included in this published article [and its supplementary information files].

## Abbreviations

AAO: American Academy of Ophthalmology
Aβ: β-amyloid
AD: Alzheimer’s disease
AMD: Age-related macular degeneration
AUC: Area under the curve
BP: Biological processes
CC: Cellular components
CCT2: Chaperonin containing TCP1 subunit 2
CMA: Chaperone-mediated autophagy
CNV: Choroidal neovascular membrane
EMT: Epithelial-mesenchymal transition
GA: Geographic atrophy
GO: Gene Ontology
KEGG: Kyoto Encyclopedia of Genes and Genomes
MF: Molecular functions
MSigDB: Molecular Signatures Database
ROC: Receiver operating characteristic
RPE: Retinal pigment epithelium
ssGSEA: Single-sample gene set enrichment analysis
