# Supplementary material for: Clinical characterization of CCT2 and its role in autophagy regulation during age-related macular degeneration
Source: Sci Rep. 2025 May 15;15:16849. doi: 10.1038/s41598-025-01907-1 (PMC12081695; doi:10.1038/s41598-025-01907-1)
Supplement: Supplementary file 1 — Supplementary Material 1 [file 41598_2025_1907_MOESM1_ESM.pdf]

Supplementary figure 1

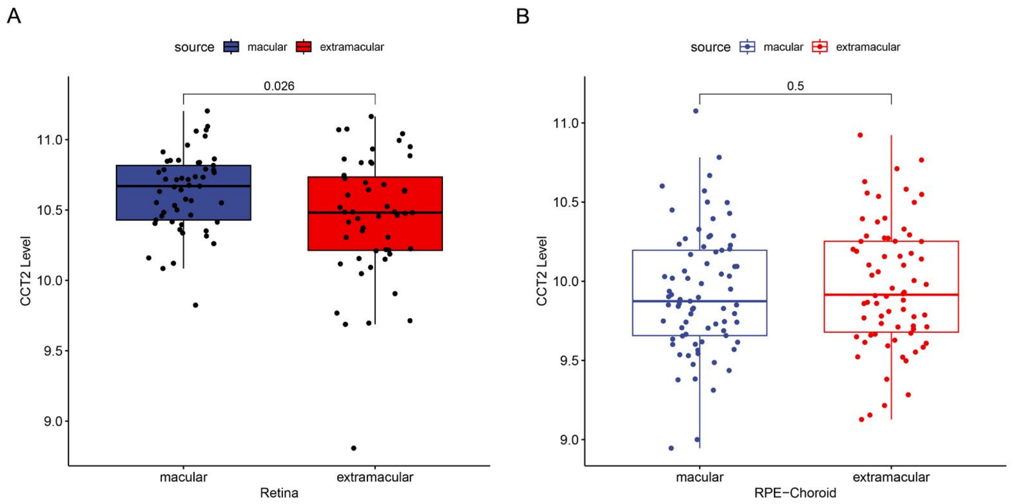

Supplementary figure 1 The chaperonin containing TCP1 subunit 2 (CCT2) expression level between macular and extramacular region within the same tissue. (A) Retina. (B) RPE-choroid.

Supplementary figure 2

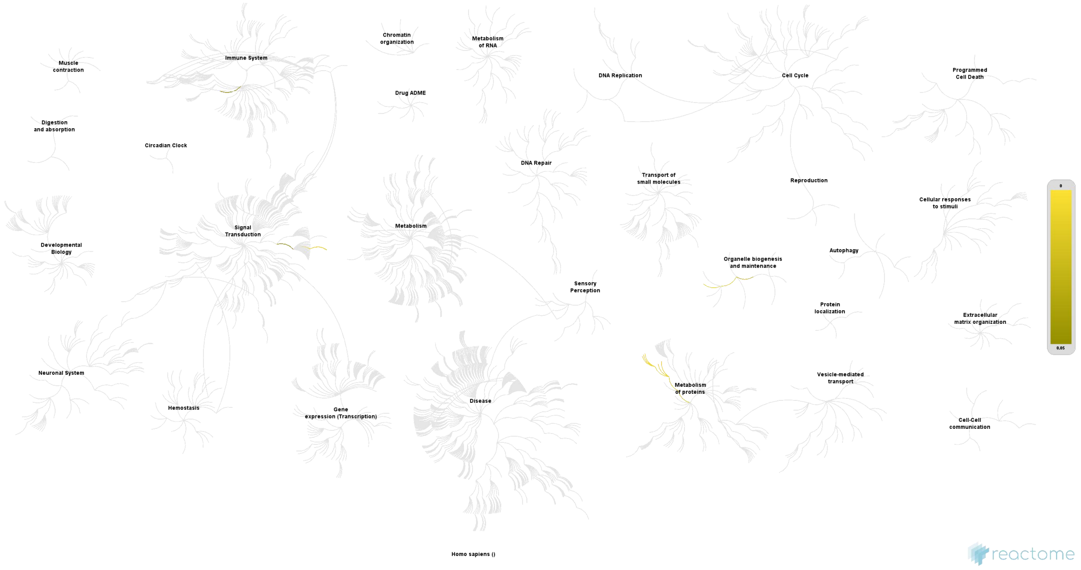

Supplementary figure 2 A genome-wide analysis of the chaperonin containing TCP1 subunit 2 (CCT2) Reactome pathway. Colors from light to dark represent p values from small to large.

Supplementary Table 1 386 genes correlated with CCT2 in the macular region

Genes	R	pvalue	Regulation
CCT2	1	0	postive
RNF14	7.97E-01	4.74E-08	postive
ANXA7	7.78E-01	1.63E-07	postive
SAE1	7.73E-01	2.16E-07	postive
VEGFA	-7.72E-01	2.32E-07	negative
NAP1L5	7.55E-01	6.05E-07	postive
TIMM17A	7.51E-01	7.25E-07	postive
GNG2	7.49E-01	8.02E-07	postive
NAT5	7.46E-01	9.53E-07	postive
CHP	7.43E-01	1.12E-06	postive
ACAT1	7.39E-01	1.35E-06	postive
PIGB	7.35E-01	1.64E-06	postive
ZFYVE27	7.29E-01	2.19E-06	postive
RXRA	-7.27E-01	2.50E-06	negative
ANGPTL4	-7.19E-01	3.49E-06	negative
FAM82A2	7.18E-01	3.78E-06	postive
CYB5R4	7.16E-01	4.05E-06	postive
LOC100128439	-7.15E-01	4.17E-06	negative
TXNDC13	7.10E-01	5.23E-06	postive
C10orf10	-7.10E-01	5.27E-06	negative
WDR77	7.08E-01	5.71E-06	postive
FBXL5	7.08E-01	5.74E-06	postive
VPS45	7.07E-01	5.97E-06	postive
LIPT1	7.06E-01	6.39E-06	postive
CXXC5	-7.03E-01	7.14E-06	negative
C18orf10	7.00E-01	8.13E-06	postive
SAP30	-6.99E-01	8.55E-06	negative
ABHD7	6.98E-01	8.99E-06	postive
FCER1G	-6.92E-01	1.13E-05	negative
LOC152217	6.91E-01	1.19E-05	postive
USP46	6.91E-01	1.22E-05	postive
SH3GL2	6.87E-01	1.41E-05	postive
TM9SF2	6.86E-01	1.47E-05	postive
RNMTL1	6.85E-01	1.54E-05	postive
IGFBP5	-6.84E-01	1.60E-05	negative
SLC2A1	-6.84E-01	1.61E-05	negative
PLK2	6.82E-01	1.70E-05	postive
P4HA1	-6.82E-01	1.72E-05	negative
CFL2	6.82E-01	1.75E-05	postive
VPS4B	6.81E-01	1.76E-05	postive
MTMR9	6.80E-01	1.88E-05	postive
DIRAS2	6.78E-01	2.04E-05	postive

PSEN2	6.77E-01	2.07E-05	postive
EHD2	-6.77E-01	2.10E-05	negative
POLR2B	6.76E-01	2.18E-05	postive
INPP5A	6.75E-01	2.24E-05	postive
ADM	-6.72E-01	2.55E-05	negative
GLRX5	6.70E-01	2.73E-05	postive
SUSD1	6.68E-01	2.94E-05	postive
AK3L1	-6.67E-01	3.07E-05	negative
DYNC1LI1	6.67E-01	3.07E-05	postive
IRF7	-6.66E-01	3.18E-05	negative
SRPRB	6.66E-01	3.18E-05	postive
TAGLN3	6.66E-01	3.23E-05	postive
PSMC1	6.65E-01	3.26E-05	postive
GTPBP2	-6.65E-01	3.27E-05	negative
USP8	6.64E-01	3.41E-05	postive
PPP1R3B	-6.64E-01	3.42E-05	negative
FES	-6.64E-01	3.43E-05	negative
UROS	6.64E-01	3.45E-05	postive
KIAA0317	6.63E-01	3.59E-05	postive
TGM2	-6.63E-01	3.61E-05	negative
SRCRB4D	6.62E-01	3.72E-05	postive
CYP4X1	6.61E-01	3.81E-05	postive
BAI3	6.61E-01	3.86E-05	postive
LANCL1	6.60E-01	3.97E-05	postive
CCT6P1	6.60E-01	4.02E-05	postive
LILRB3	-6.57E-01	4.41E-05	negative
CLTC	6.57E-01	4.43E-05	postive
TOR1A	6.57E-01	4.47E-05	postive
SLC39A14	-6.56E-01	4.60E-05	negative
EPS8L2	-6.54E-01	4.90E-05	negative
CMPK1	6.54E-01	4.96E-05	postive
TIMM23	6.54E-01	4.99E-05	postive
MTERFD3	6.53E-01	5.05E-05	postive
CDS2	6.53E-01	5.14E-05	postive
PPP2CA	6.52E-01	5.27E-05	postive
YARS2	6.52E-01	5.28E-05	postive
MLX	6.51E-01	5.39E-05	postive
SLC25A20	6.51E-01	5.43E-05	postive
SLC6A12	-6.51E-01	5.43E-05	negative
SH3BGRL2	6.51E-01	5.50E-05	postive
MTCH1	6.51E-01	5.51E-05	postive
FOSL2	-6.51E-01	5.54E-05	negative
LOC344595	6.49E-01	5.88E-05	postive
KCNC2	6.48E-01	5.97E-05	postive

CDC37L1	6.48E-01	5.98E-05	postive
IMPAD1	6.48E-01	6.07E-05	postive
MRLC2	6.48E-01	6.11E-05	postive
GOT1	6.47E-01	6.21E-05	postive
LRRC49	6.47E-01	6.36E-05	postive
TIMM22	6.46E-01	6.52E-05	postive
PPP1R13L	-6.46E-01	6.53E-05	negative
ARRDC2	-6.44E-01	6.89E-05	negative
GBP2	-6.44E-01	7.01E-05	negative
PNMA3	6.43E-01	7.11E-05	postive
HIG2	-6.43E-01	7.24E-05	negative
FBXO42	-6.41E-01	7.76E-05	negative
ATP6V1G2	6.40E-01	8.06E-05	postive
C16orf88	6.39E-01	8.19E-05	postive
KLHL34	-6.39E-01	8.32E-05	negative
PAK6	6.39E-01	8.39E-05	postive
SLC17A8	6.38E-01	8.56E-05	postive
MAFF	-6.37E-01	8.90E-05	negative
GDF15	-6.37E-01	8.90E-05	negative
HIST1H1D	-6.37E-01	8.96E-05	negative
RCN2	6.36E-01	9.11E-05	postive
RNF220	6.36E-01	9.13E-05	postive
GABRG2	6.36E-01	9.22E-05	postive
NTRK1	6.34E-01	9.91E-05	postive
ZNF627	6.33E-01	9.97E-05	postive
C1orf162	-6.33E-01	1.00E-04	negative
ARL1	6.33E-01	1.01E-04	postive
PABPC1	-6.33E-01	1.02E-04	negative
POP7	6.33E-01	1.02E-04	postive
IMMT	6.32E-01	1.04E-04	postive
MAP2K6	6.31E-01	1.07E-04	postive
PFKFB4	-6.31E-01	1.08E-04	negative
SLPI	-6.30E-01	1.11E-04	negative
TRIM37	6.30E-01	1.11E-04	postive
ANKHD1-EIF4EBP3	-6.30E-01	1.12E-04	negative
TSPYL5	6.30E-01	1.13E-04	postive
STRAP	6.29E-01	1.16E-04	postive
TBCE	6.29E-01	1.16E-04	postive
CYP1B1	-6.29E-01	1.17E-04	negative
MAGEE1	6.28E-01	1.18E-04	postive
ATXN7	-6.28E-01	1.19E-04	negative
DCTPP1	6.28E-01	1.21E-04	postive
ITFG1	6.27E-01	1.22E-04	postive
DHX36	6.27E-01	1.23E-04	postive

BBX	-6.26E-01	1.26E-04	negative
PSMD5	6.26E-01	1.27E-04	postive
TPI1	-6.25E-01	1.31E-04	negative
SUCLA2	6.25E-01	1.33E-04	postive
GNL3	6.25E-01	1.33E-04	postive
POU4F1	6.24E-01	1.34E-04	postive
TCERG1L	6.23E-01	1.39E-04	postive
FLJ40142	6.23E-01	1.41E-04	postive
FLJ10038	6.22E-01	1.43E-04	postive
FAM92A1	6.21E-01	1.51E-04	postive
OPA1	6.20E-01	1.55E-04	postive
KIAA0644	6.19E-01	1.59E-04	postive
SLC25A5	6.19E-01	1.60E-04	postive
THTPA	6.19E-01	1.60E-04	postive
UXS1	6.19E-01	1.61E-04	postive
VDAC3	6.18E-01	1.62E-04	postive
ZNF395	-6.18E-01	1.63E-04	negative
PSMD14	6.18E-01	1.64E-04	postive
SLC11A1	-6.18E-01	1.65E-04	negative
SYT13	6.18E-01	1.66E-04	postive
GPRASP2	6.18E-01	1.66E-04	postive
SH3GL3	6.17E-01	1.67E-04	postive
MRPL39	6.17E-01	1.69E-04	postive
SC5DL	6.17E-01	1.70E-04	postive
ADAM8	-6.17E-01	1.70E-04	negative
FAM162A	-6.17E-01	1.70E-04	negative
ADRA2C	-6.17E-01	1.71E-04	negative
ATP6V1A	6.16E-01	1.73E-04	postive
LRRN3	6.16E-01	1.73E-04	postive
ENOX2	6.16E-01	1.74E-04	postive
LOC257396	6.16E-01	1.74E-04	postive
RBMX2	6.16E-01	1.75E-04	postive
CA9	-6.15E-01	1.82E-04	negative
JMJD6	-6.14E-01	1.88E-04	negative
B4GALT1	-6.13E-01	1.91E-04	negative
IFITM2	-6.13E-01	1.93E-04	negative
NDUFA8	6.12E-01	1.96E-04	postive
LEPROTL1	6.11E-01	2.01E-04	postive
LDHB	6.11E-01	2.01E-04	postive
MIA3	6.11E-01	2.05E-04	postive
HK2	-6.11E-01	2.05E-04	negative
PXN	-6.11E-01	2.06E-04	negative
KCNAB1	6.10E-01	2.08E-04	postive
PFKM	6.10E-01	2.08E-04	postive

SRGN	-6.10E-01	2.10E-04	negative
MYF6	6.10E-01	2.12E-04	postive
DOLK	6.10E-01	2.13E-04	postive
SLC6A1	6.08E-01	2.22E-04	postive
OSTM1	6.08E-01	2.24E-04	postive
ACTR10	6.08E-01	2.25E-04	postive
IFITM4P	-6.07E-01	2.30E-04	negative
TUSC2	6.07E-01	2.30E-04	postive
PNMA1	6.07E-01	2.31E-04	postive
EPB41L3	6.07E-01	2.32E-04	postive
SLC39A2	6.06E-01	2.34E-04	postive
TMEM41B	6.06E-01	2.38E-04	postive
SERTAD4	6.06E-01	2.39E-04	postive
ATP1B1	6.06E-01	2.40E-04	postive
COPG	6.05E-01	2.41E-04	postive
LDOC1L	6.05E-01	2.48E-04	postive
ZNF57	6.04E-01	2.50E-04	postive
ABHD14B	-6.04E-01	2.52E-04	negative
MST150	-6.04E-01	2.54E-04	negative
KIAA0652	-6.04E-01	2.55E-04	negative
MFSD4	6.03E-01	2.56E-04	postive
ANG	-6.03E-01	2.56E-04	negative
RELA	-6.03E-01	2.57E-04	negative
CTPS	6.03E-01	2.57E-04	postive
REEP5	6.03E-01	2.58E-04	postive
C5orf44	6.03E-01	2.63E-04	postive
PTDSS1	6.01E-01	2.76E-04	postive
PPARGC1A	6.00E-01	2.81E-04	postive
ATP5O	6.00E-01	2.82E-04	postive
POLR3F	6.00E-01	2.85E-04	postive
C1QBP	6.00E-01	2.85E-04	postive
DYRK2	-6.00E-01	2.87E-04	negative
WDR12	5.99E-01	2.90E-04	postive
SMS	5.99E-01	2.95E-04	postive
TIGD5	5.98E-01	3.02E-04	postive
MRPS15	5.98E-01	3.04E-04	postive
DDHD2	5.97E-01	3.07E-04	postive
IL6R	-5.97E-01	3.12E-04	negative
C10orf137	5.97E-01	3.14E-04	postive
IMPA1	5.96E-01	3.16E-04	postive
JUN	-5.96E-01	3.17E-04	negative
MLLT11	5.96E-01	3.17E-04	postive
GLCCI1	-5.95E-01	3.27E-04	negative
PSMB7	5.95E-01	3.27E-04	postive

OCIAD1	5.95E-01	3.28E-04	postive
LOC338756	5.94E-01	3.37E-04	postive
ACTN1	-5.94E-01	3.38E-04	negative
GMFB	5.94E-01	3.40E-04	postive
TNFRSF10B	-5.94E-01	3.42E-04	negative
LOC145783	5.93E-01	3.43E-04	postive
CCDC56	5.93E-01	3.47E-04	postive
KLHDC5	5.93E-01	3.49E-04	postive
PPP2R3C	5.92E-01	3.53E-04	postive
PLOD2	-5.92E-01	3.53E-04	negative
CETN2	5.92E-01	3.54E-04	postive
ACOT7	5.92E-01	3.54E-04	postive
CNNM3	-5.92E-01	3.56E-04	negative
CGREF1	5.92E-01	3.56E-04	postive
HNRNPA1	5.91E-01	3.67E-04	postive
DCUN1D2	5.90E-01	3.82E-04	postive
PAWR	-5.90E-01	3.84E-04	negative
ADRB2	5.89E-01	3.94E-04	postive
PSME2	-5.88E-01	3.98E-04	negative
DEM1	5.88E-01	3.99E-04	postive
TRUB1	5.88E-01	4.00E-04	postive
AMACR	5.88E-01	4.02E-04	postive
GALNT10	-5.87E-01	4.09E-04	negative
EXOC1	5.87E-01	4.09E-04	postive
FBXO3	5.87E-01	4.11E-04	postive
TM2D2	5.87E-01	4.13E-04	postive
NKX3-1	5.87E-01	4.14E-04	postive
EIF5	5.87E-01	4.17E-04	postive
GPR84	-5.87E-01	4.17E-04	negative
HINT1	5.86E-01	4.21E-04	postive
BCL3	-5.86E-01	4.23E-04	negative
TMEM30A	5.86E-01	4.28E-04	postive
CD86	-5.86E-01	4.30E-04	negative
COQ3	5.85E-01	4.34E-04	postive
UBAC1	5.85E-01	4.34E-04	postive
SLBP	5.85E-01	4.39E-04	postive
FOXO1	-5.85E-01	4.42E-04	negative
PKIB	5.84E-01	4.44E-04	postive
PPIA	5.84E-01	4.44E-04	postive
SNX13	5.84E-01	4.47E-04	postive
LOC728324	5.84E-01	4.49E-04	postive
LOC399491	-5.84E-01	4.50E-04	negative
S1PR2	-5.84E-01	4.50E-04	negative
C9orf125	5.84E-01	4.56E-04	postive

MCF2	5.84E-01	4.56E-04	postive
SRPK2	5.83E-01	4.57E-04	postive
AIFM3	5.83E-01	4.59E-04	postive
LIMS1	-5.81E-01	4.83E-04	negative
FAM134B	5.81E-01	4.87E-04	postive
SYNGR3	5.81E-01	4.93E-04	postive
C7orf54	-5.81E-01	4.95E-04	negative
TMEM200A	5.80E-01	4.99E-04	postive
LYPD6B	5.80E-01	5.00E-04	postive
KIAA1219	5.80E-01	5.04E-04	postive
FKBP11	-5.80E-01	5.06E-04	negative
FPR1	-5.79E-01	5.13E-04	negative
UCHL1	5.79E-01	5.14E-04	postive
CASD1	5.79E-01	5.23E-04	postive
STRC	-5.78E-01	5.26E-04	negative
VGLL3	5.78E-01	5.31E-04	postive
RHOG	-5.78E-01	5.36E-04	negative
ADPRHL1	5.77E-01	5.39E-04	postive
CABIN1	-5.77E-01	5.53E-04	negative
GBAS	5.76E-01	5.54E-04	postive
KIAA1618	-5.76E-01	5.67E-04	negative
PXDNL	5.75E-01	5.70E-04	postive
ABCC6	5.75E-01	5.71E-04	postive
COMMD4	5.75E-01	5.72E-04	postive
ASPHD1	5.75E-01	5.79E-04	postive
UTP18	5.74E-01	5.87E-04	postive
ATF3	-5.74E-01	5.92E-04	negative
SPOCD1	-5.74E-01	5.98E-04	negative
GSTA4	5.73E-01	6.02E-04	postive
ABAT	5.73E-01	6.03E-04	postive
MRPS27	5.73E-01	6.05E-04	postive
CEBPD	-5.73E-01	6.05E-04	negative
LGALS3BP	-5.73E-01	6.15E-04	negative
SDC2	-5.72E-01	6.19E-04	negative
SRFBP1	5.72E-01	6.23E-04	postive
ZNF134	5.72E-01	6.23E-04	postive
GPC3	5.72E-01	6.24E-04	postive
CRYBB3	5.71E-01	6.39E-04	postive
MAK16	5.71E-01	6.39E-04	postive
VISA	-5.71E-01	6.42E-04	negative
PXMP3	5.70E-01	6.55E-04	postive
YEATS2	-5.70E-01	6.59E-04	negative
PRDM13	5.69E-01	6.72E-04	postive
NDUFS5	5.69E-01	6.76E-04	postive

RWDD2B	5.69E-01	6.78E-04	postive
BTBD10	5.69E-01	6.78E-04	postive
CCDC44	5.69E-01	6.83E-04	postive
PELI2	5.68E-01	6.89E-04	postive
BEX2	5.68E-01	6.91E-04	postive
LOC100134167	5.68E-01	6.98E-04	postive
PRPSAP1	5.68E-01	7.02E-04	postive
FKBP9	5.68E-01	7.03E-04	postive
GCSH	5.68E-01	7.03E-04	postive
REPS2	5.67E-01	7.07E-04	postive
YRDC	5.67E-01	7.11E-04	postive
UBQLN1	5.67E-01	7.12E-04	postive
EBF1	5.66E-01	7.26E-04	postive
LRRC8B	5.66E-01	7.29E-04	postive
MEX3B	5.66E-01	7.29E-04	postive
SYT4	5.66E-01	7.30E-04	postive
MTF1	-5.66E-01	7.42E-04	negative
C7orf28B	5.65E-01	7.51E-04	postive
SLC35A1	5.65E-01	7.61E-04	postive
MRPL45	5.64E-01	7.68E-04	postive
NPM2	5.64E-01	7.75E-04	postive
PPP2R1A	5.64E-01	7.77E-04	postive
TMEM62	5.64E-01	7.77E-04	postive
LSM14B	5.64E-01	7.78E-04	postive
IL6	-5.63E-01	7.92E-04	negative
TUBB3	5.63E-01	7.96E-04	postive
MAP3K6	-5.63E-01	7.98E-04	negative
PRPS1	5.63E-01	7.99E-04	postive
SHMT2	-5.62E-01	8.06E-04	negative
HOXB2	-5.62E-01	8.11E-04	negative
CRHR1	5.62E-01	8.12E-04	postive
PRKRIP1	-5.62E-01	8.15E-04	negative
OAT	5.62E-01	8.22E-04	postive
SCN4B	5.62E-01	8.23E-04	postive
GADD45B	-5.62E-01	8.23E-04	negative
CHMP2B	5.62E-01	8.24E-04	postive
LOC389895	5.61E-01	8.40E-04	postive
GTF3C3	5.61E-01	8.40E-04	postive
C8orf58	-5.61E-01	8.46E-04	negative
EPDR1	5.60E-01	8.52E-04	postive
BACE2	-5.60E-01	8.53E-04	negative
SLC25A46	5.60E-01	8.54E-04	postive
FLJ11151	5.60E-01	8.55E-04	postive
PNMA5	5.60E-01	8.56E-04	postive

MFF	5.59E-01	8.72E-04	postive
PRRT3	5.59E-01	8.76E-04	postive
GFM1	5.59E-01	8.82E-04	postive
FAM158A	-5.59E-01	8.84E-04	negative
C6orf168	5.59E-01	8.91E-04	postive
MARCKS	5.59E-01	8.93E-04	postive
C11orf1	5.58E-01	9.05E-04	postive
SCFD2	5.58E-01	9.08E-04	postive
C4orf49	5.58E-01	9.09E-04	postive
SAV1	-5.57E-01	9.21E-04	negative
FREQ	5.57E-01	9.23E-04	postive
NDUFB8	5.57E-01	9.30E-04	postive
AFAP1L1	-5.57E-01	9.30E-04	negative
SPIRE1	5.57E-01	9.39E-04	postive
PPIAL4A	5.56E-01	9.41E-04	postive
KCNE4	-5.56E-01	9.43E-04	negative
HDHD2	5.56E-01	9.46E-04	postive
AZI2	5.56E-01	9.46E-04	postive
SLC26A6	-5.56E-01	9.47E-04	negative
RAB38	-5.56E-01	9.48E-04	negative
NPR3	5.56E-01	9.51E-04	postive
IER3	-5.56E-01	9.56E-04	negative
XK	5.56E-01	9.62E-04	postive
SLC35F1	5.55E-01	9.73E-04	postive
IRF1	-5.55E-01	9.74E-04	negative
TSKU	-5.55E-01	9.75E-04	negative
C21orf33	5.55E-01	9.79E-04	postive
IER5L	-5.55E-01	9.82E-04	negative
ILF2	5.55E-01	9.83E-04	postive
RAC2	-5.55E-01	9.88E-04	negative
KIFAP3	5.54E-01	9.90E-04	postive
NEFL	5.54E-01	9.91E-04	postive
STMN3	5.54E-01	9.97E-04	postive
EIF3K	5.54E-01	9.97E-04	postive
JARID1A	-5.54E-01	9.97E-04	negative
LOC100132763	-5.54E-01	9.99E-04	negative

Supplementary Table 2   1250 genes correlated with CCT2 in the extramacular region

Genes	R	pvalue	Regulation
CCT2	1	0	postive
EID2B	6.41E-01	1.01E-04	postive
FAM162B	-6.41E-01	1.02E-04	negative
PAIP2	6.41E-01	1.03E-04	postive
ABCB10	6.41E-01	1.03E-04	postive
RBPJ	-6.41E-01	1.03E-04	negative
RPS6KA5	6.40E-01	1.04E-04	postive
SLC25A46	6.40E-01	1.05E-04	postive
VPS4B	6.40E-01	1.05E-04	postive
SLC25A3	6.40E-01	1.06E-04	postive
BAIAP2L2	-6.40E-01	1.07E-04	negative
ARFIP1	6.40E-01	1.07E-04	postive
CLDN19	-6.40E-01	1.07E-04	negative
ZNF407	6.39E-01	1.08E-04	postive
SFRS2	6.39E-01	1.08E-04	postive
LARP6	-6.39E-01	1.08E-04	negative
MIA	-6.39E-01	1.10E-04	negative
FAM135A	6.39E-01	1.10E-04	postive
C7orf28A	6.39E-01	1.10E-04	postive
PRDM4	6.39E-01	1.10E-04	postive
FANCF	6.39E-01	1.10E-04	postive
LOC727865	6.39E-01	1.10E-04	postive
TRIM63	-6.39E-01	1.10E-04	negative
MTDH	6.39E-01	1.11E-04	postive
DNAJC9	6.38E-01	1.12E-04	postive
REEP3	-6.38E-01	1.13E-04	negative
C15orf44	6.38E-01	1.13E-04	postive
C3orf75	6.38E-01	1.14E-04	postive
SSBP1	6.38E-01	1.14E-04	postive
LONRF2	6.38E-01	1.15E-04	postive
LOC100128079	-6.37E-01	1.15E-04	negative
PSMA2	6.37E-01	1.15E-04	postive
LOC220906	6.37E-01	1.15E-04	postive
TCEB2	6.37E-01	1.16E-04	postive
FXYD5	-6.37E-01	1.16E-04	negative
TGM2	-6.37E-01	1.17E-04	negative
BCAS2	6.37E-01	1.17E-04	postive
EIF4EBP1	-6.37E-01	1.18E-04	negative
TMEM66	6.37E-01	1.18E-04	postive

VSTM2A	6.36E-01	1.19E-04	postive
HIST1H2BG	-6.36E-01	1.20E-04	negative
C1orf212	6.36E-01	1.21E-04	postive
CLNS1A	6.36E-01	1.21E-04	postive
PI16	-6.36E-01	1.22E-04	negative
NUDT5	6.36E-01	1.22E-04	postive
TDRKH	6.36E-01	1.22E-04	postive
CAMK2G	6.36E-01	1.22E-04	postive
C7orf40	6.35E-01	1.23E-04	postive
ARV1	6.35E-01	1.23E-04	postive
APEH	6.35E-01	1.24E-04	postive
SFMBT1	6.35E-01	1.24E-04	postive
RFXAP	6.35E-01	1.24E-04	postive
PSMB3	6.35E-01	1.25E-04	postive
SCARNA17	-6.35E-01	1.26E-04	negative
PHLDB2	-6.35E-01	1.26E-04	negative
MORF4L1	6.35E-01	1.26E-04	postive
FAM110A	-6.34E-01	1.27E-04	negative
MBD6	-6.34E-01	1.27E-04	negative
PCDHA11	6.34E-01	1.27E-04	postive
DHTKD1	6.34E-01	1.28E-04	postive
MIF	-6.34E-01	1.28E-04	negative
PAPD4	6.34E-01	1.28E-04	postive
NCKAP1	6.34E-01	1.28E-04	postive
RPL5	6.34E-01	1.28E-04	postive
PPP1R15A	-6.34E-01	1.28E-04	negative
TMEM109	6.34E-01	1.29E-04	postive
MTA1	-6.34E-01	1.30E-04	negative
PHAX	6.33E-01	1.31E-04	postive
VASH1	-6.33E-01	1.32E-04	negative
RPS18	6.33E-01	1.34E-04	postive
SCD5	6.33E-01	1.34E-04	postive
HDAC7	-6.33E-01	1.34E-04	negative
SMS	6.32E-01	1.36E-04	postive
SYT9	6.32E-01	1.39E-04	postive
PHB2	6.32E-01	1.39E-04	postive
MAK16	6.31E-01	1.40E-04	postive
FAM158A	-6.31E-01	1.40E-04	negative
CBWD2	6.31E-01	1.41E-04	postive
WDR90	-6.31E-01	1.41E-04	negative
TNFAIP8	-6.31E-01	1.41E-04	negative
H3F3B	6.31E-01	1.41E-04	postive
ARMCX1	6.31E-01	1.43E-04	postive
LAMB2	-6.30E-01	1.45E-04	negative

NIF3L1	6.30E-01	1.46E-04	postive
RP11-129H15.2	6.30E-01	1.46E-04	postive
SLC25A36	-6.30E-01	1.47E-04	negative
ANXA7	6.30E-01	1.48E-04	postive
NUDCD1	6.30E-01	1.48E-04	postive
SUMO3	6.29E-01	1.50E-04	postive
LOC376693	-6.29E-01	1.51E-04	negative
PRRT2	-6.29E-01	1.52E-04	negative
INO80	6.28E-01	1.53E-04	postive
MECR	6.28E-01	1.54E-04	postive
IGF2AS	-6.28E-01	1.54E-04	negative
LONRF1	6.28E-01	1.55E-04	postive
MTUS1	6.28E-01	1.56E-04	postive
SIRT1	6.28E-01	1.56E-04	postive
OCIAD2	6.28E-01	1.56E-04	postive
LOC131055	6.28E-01	1.56E-04	postive
SSU72	6.28E-01	1.57E-04	postive
STX4	-6.28E-01	1.58E-04	negative
APEX1	6.27E-01	1.58E-04	postive
FAM18B	6.27E-01	1.58E-04	postive
YRDC	6.27E-01	1.58E-04	postive
AK3L1	-6.27E-01	1.59E-04	negative
NME7	6.27E-01	1.59E-04	postive
MYO1F	-6.27E-01	1.59E-04	negative
RNF24	-6.27E-01	1.61E-04	negative
TMEM126B	6.27E-01	1.61E-04	postive
ANKK1	-6.27E-01	1.61E-04	negative
C13orf23	6.27E-01	1.62E-04	postive
BEX4	6.27E-01	1.62E-04	postive
KRT18	-6.27E-01	1.63E-04	negative
ADRA2C	-6.26E-01	1.63E-04	negative
TLE3	-6.26E-01	1.64E-04	negative
DGCR6L	6.26E-01	1.65E-04	postive
SELI	6.26E-01	1.65E-04	postive
BATF3	-6.26E-01	1.65E-04	negative
PHF10	-6.26E-01	1.66E-04	negative
SNX4	6.26E-01	1.66E-04	postive
CBX1	-6.26E-01	1.67E-04	negative
LIMA1	6.26E-01	1.68E-04	postive
PPP1R10	6.26E-01	1.68E-04	postive
PRKAR1A	6.25E-01	1.68E-04	postive
LOC728380	6.25E-01	1.69E-04	postive
ATP6V1G1	6.25E-01	1.69E-04	postive
BAI3	6.25E-01	1.70E-04	postive

GALNT10	-6.24E-01	1.74E-04	negative
C11orf31	6.24E-01	1.74E-04	postive
LOC645884	6.24E-01	1.75E-04	postive
GOLGA7	6.24E-01	1.76E-04	postive
KIF1C	-6.24E-01	1.78E-04	negative
DAZAP2	6.23E-01	1.82E-04	postive
C5orf30	6.23E-01	1.83E-04	postive
POU2AF1	-6.23E-01	1.83E-04	negative
ZNF3	6.23E-01	1.83E-04	postive
TIMM23	6.23E-01	1.84E-04	postive
LCMT2	6.22E-01	1.85E-04	postive
EGFL6	-6.22E-01	1.85E-04	negative
POLR3B	6.22E-01	1.86E-04	postive
ATMIN	6.22E-01	1.87E-04	postive
FOSL2	-6.22E-01	1.87E-04	negative
TMEM209	6.22E-01	1.88E-04	postive
VTI1B	6.22E-01	1.89E-04	postive
TUB	6.21E-01	1.90E-04	postive
GTF2F2	6.21E-01	1.91E-04	postive
MAGED2	6.21E-01	1.92E-04	postive
IFT80	6.21E-01	1.93E-04	postive
PRPS1	6.21E-01	1.94E-04	postive
SFRS3	6.21E-01	1.95E-04	postive
RNF149	6.21E-01	1.95E-04	postive
NAV2	-6.21E-01	1.96E-04	negative
PRSS35	6.20E-01	1.96E-04	postive
RNF138	6.20E-01	1.97E-04	postive
TSGA14	6.20E-01	1.98E-04	postive
ECHDC1	6.20E-01	1.99E-04	postive
C18orf55	6.20E-01	1.99E-04	postive
LSG1	6.20E-01	2.00E-04	postive
PAFAH1B2	6.20E-01	2.01E-04	postive
C12orf47	6.20E-01	2.01E-04	postive
SSH1	-6.20E-01	2.01E-04	negative
RSRC2	6.20E-01	2.01E-04	postive
BBS10	6.20E-01	2.01E-04	postive
ZNF624	6.20E-01	2.02E-04	postive
KCTD16	-6.20E-01	2.02E-04	negative
RBM45	6.19E-01	2.02E-04	postive
MECP2	-6.19E-01	2.03E-04	negative
BCKDHA	-6.19E-01	2.04E-04	negative
TM7SF3	6.19E-01	2.04E-04	postive
NOX4	6.19E-01	2.04E-04	postive
SLBP	6.19E-01	2.05E-04	postive

NUDT6	6.19E-01	2.06E-04	postive
MRPL10	6.19E-01	2.06E-04	postive
C2orf64	6.19E-01	2.06E-04	postive
KIAA0999	6.19E-01	2.06E-04	postive
SPATA5L1	6.19E-01	2.07E-04	postive
BACE2	-6.19E-01	2.07E-04	negative
DPYSL4	-6.19E-01	2.07E-04	negative
LZTFL1	6.19E-01	2.08E-04	postive
RPA3	6.18E-01	2.09E-04	postive
GRHPR	6.18E-01	2.10E-04	postive
KIAA1012	6.18E-01	2.10E-04	postive
SEC31B	-6.18E-01	2.11E-04	negative
WIF1	6.18E-01	2.11E-04	postive
LYSMD2	6.18E-01	2.12E-04	postive
CRBN	6.18E-01	2.13E-04	postive
NDUFAF1	6.17E-01	2.15E-04	postive
TMEM138	6.17E-01	2.16E-04	postive
SON	6.17E-01	2.17E-04	postive
NDUFA5	6.17E-01	2.17E-04	postive
AP1M1	6.17E-01	2.17E-04	postive
GNPTG	6.17E-01	2.19E-04	postive
SLC7A5	-6.16E-01	2.22E-04	negative
RALA	6.16E-01	2.23E-04	postive
EIF4A2	6.16E-01	2.24E-04	postive
EHD2	-6.16E-01	2.25E-04	negative
CTR9	6.16E-01	2.26E-04	postive
PDCD6IP	6.16E-01	2.27E-04	postive
TP53BP2	6.16E-01	2.27E-04	postive
IL6	-6.16E-01	2.28E-04	negative
PQLC1	-6.15E-01	2.30E-04	negative
SEPX1	6.15E-01	2.32E-04	postive
HDAC2	6.15E-01	2.32E-04	postive
KIAA1468	6.15E-01	2.33E-04	postive
CCDC34	6.15E-01	2.34E-04	postive
LMBRD1	6.15E-01	2.34E-04	postive
RPL23	-6.15E-01	2.35E-04	negative
UNK	-6.14E-01	2.35E-04	negative
INTS10	6.14E-01	2.39E-04	postive
C4orf27	6.14E-01	2.41E-04	postive
COG8	6.14E-01	2.42E-04	postive
DYNLL1	6.13E-01	2.43E-04	postive
LOC100128822	6.13E-01	2.46E-04	postive
CYB5R4	6.13E-01	2.46E-04	postive
FAM3C	6.13E-01	2.48E-04	postive

PSMG1	6.13E-01	2.49E-04	postive
ASH2L	6.13E-01	2.49E-04	postive
CDK7	6.13E-01	2.50E-04	postive
MED9	6.12E-01	2.50E-04	postive
LOC100130289	6.12E-01	2.53E-04	postive
DIRAS1	-6.12E-01	2.53E-04	negative
CHD6	6.12E-01	2.54E-04	postive
SMARCAD1	6.12E-01	2.55E-04	postive
FAM119A	-6.12E-01	2.55E-04	negative
KIN	6.12E-01	2.56E-04	postive
ZMIZ2	-6.11E-01	2.59E-04	negative
STAB1	-6.11E-01	2.60E-04	negative
REV3L	6.11E-01	2.60E-04	postive
C12orf43	6.11E-01	2.61E-04	postive
TIMP2	-6.11E-01	2.61E-04	negative
UGP2	6.11E-01	2.62E-04	postive
TMEM106B	6.11E-01	2.62E-04	postive
C2orf44	6.11E-01	2.62E-04	postive
LOC100131482	6.11E-01	2.64E-04	postive
ADAMTS9	-6.11E-01	2.65E-04	negative
PREB	6.10E-01	2.68E-04	postive
SYT4	6.10E-01	2.69E-04	postive
LRPPRC	6.10E-01	2.69E-04	postive
LOC344595	6.10E-01	2.70E-04	postive
CLN6	6.10E-01	2.71E-04	postive
LAGE3	6.09E-01	2.74E-04	postive
HK2	-6.09E-01	2.74E-04	negative
TNNC1	-6.09E-01	2.74E-04	negative
TWF1	6.09E-01	2.76E-04	postive
LRRC8B	6.09E-01	2.78E-04	postive
RTCD1	6.09E-01	2.80E-04	postive
SETBP1	-6.09E-01	2.81E-04	negative
LYPLAL1	6.09E-01	2.81E-04	postive
TSEN2	6.08E-01	2.81E-04	postive
RINT1	6.08E-01	2.82E-04	postive
HTRA4	-6.08E-01	2.83E-04	negative
P2RX7	-6.08E-01	2.83E-04	negative
EID2	6.08E-01	2.85E-04	postive
FGF13	6.08E-01	2.85E-04	postive
JAG1	6.08E-01	2.87E-04	postive
NCRNA00152	-6.08E-01	2.87E-04	negative
FAM120AOS	6.08E-01	2.89E-04	postive
RPL26L1	6.07E-01	2.91E-04	postive
GABRR1	6.07E-01	2.91E-04	postive

KIAA1618	-6.07E-01	2.92E-04	negative
ESF1	6.07E-01	2.93E-04	postive
SLC30A5	6.07E-01	2.93E-04	postive
PIGX	6.07E-01	2.95E-04	postive
MAGI2	6.07E-01	2.97E-04	postive
USP16	6.06E-01	2.99E-04	postive
SMEK2	6.06E-01	3.02E-04	postive
SLC35F1	6.06E-01	3.03E-04	postive
ZNF354A	6.06E-01	3.05E-04	postive
ST3GAL5	-6.06E-01	3.05E-04	negative
SF3A2	-6.06E-01	3.06E-04	negative
DEK	6.06E-01	3.07E-04	postive
IMPG1	6.05E-01	3.08E-04	postive
CHCHD1	6.05E-01	3.08E-04	postive
BSCL2	6.05E-01	3.10E-04	postive
HDHD2	6.05E-01	3.13E-04	postive
SIRPA	-6.05E-01	3.15E-04	negative
TUSC1	6.05E-01	3.16E-04	postive
TNS1	-6.04E-01	3.17E-04	negative
KRT18P33	-6.04E-01	3.19E-04	negative
TRIP4	6.04E-01	3.20E-04	postive
C11orf9	-6.04E-01	3.25E-04	negative
ZNF518A	6.03E-01	3.28E-04	postive
C12orf29	6.03E-01	3.28E-04	postive
THBD	-6.03E-01	3.29E-04	negative
TEP1	-6.03E-01	3.29E-04	negative
ZNHIT3	6.03E-01	3.29E-04	postive
COX4I1	6.03E-01	3.30E-04	postive
C19orf68	-6.03E-01	3.30E-04	negative
HIG2	-6.03E-01	3.31E-04	negative
CTSC	-6.03E-01	3.34E-04	negative
FEZ1	6.02E-01	3.40E-04	postive
LZTS2	-6.02E-01	3.41E-04	negative
C2orf68	6.02E-01	3.43E-04	postive
ERRFI1	6.02E-01	3.44E-04	postive
ZHX1	6.01E-01	3.45E-04	postive
IL6R	-6.01E-01	3.46E-04	negative
MSRB2	6.01E-01	3.49E-04	postive
TMSB15B	6.01E-01	3.50E-04	postive
AGK	6.01E-01	3.51E-04	postive
ETV6	-6.01E-01	3.51E-04	negative
IGFALS	-6.01E-01	3.52E-04	negative
PPP1R13L	-6.01E-01	3.53E-04	negative
C6orf64	6.00E-01	3.58E-04	postive

GNPAT	6.00E-01	3.62E-04	postive
TMEM108	6.00E-01	3.64E-04	postive
RNF170	6.00E-01	3.64E-04	postive
BIVM	5.99E-01	3.65E-04	postive
FKSG24	5.99E-01	3.66E-04	postive
FAM84B	5.99E-01	3.67E-04	postive
BRSK2	-5.99E-01	3.70E-04	negative
GTF3C2	5.99E-01	3.71E-04	postive
LPPR4	5.99E-01	3.71E-04	postive
PCYOX1	5.99E-01	3.72E-04	postive
ANGPTL4	-5.98E-01	3.78E-04	negative
C3orf63	5.98E-01	3.80E-04	postive
GLRX5	5.98E-01	3.80E-04	postive
PSMD10	5.98E-01	3.82E-04	postive
PRKAB1	5.98E-01	3.82E-04	postive
LOC149501	-5.98E-01	3.83E-04	negative
DEM1	5.98E-01	3.83E-04	postive
MAGEL2	5.98E-01	3.84E-04	postive
GNG13	-5.98E-01	3.85E-04	negative
ZCCHC8	5.97E-01	3.87E-04	postive
AHCYL1	5.97E-01	3.88E-04	postive
TTC15	5.97E-01	3.88E-04	postive
SERPINH1	-5.97E-01	3.89E-04	negative
SLC25A22	-5.97E-01	3.89E-04	negative
MRPL20	5.97E-01	3.89E-04	postive
LOC25845	5.97E-01	3.90E-04	postive
UMPS	5.97E-01	3.91E-04	postive
LSM3	5.97E-01	3.92E-04	postive
PRX	-5.97E-01	3.94E-04	negative
OTUD1	5.97E-01	3.94E-04	postive
DGCR8	-5.97E-01	3.96E-04	negative
SLITRK2	-5.96E-01	3.98E-04	negative
CAT	5.96E-01	3.98E-04	postive
EEF1G	5.96E-01	4.00E-04	postive
WDR22	-5.96E-01	4.00E-04	negative
RBBP4	5.96E-01	4.02E-04	postive
GFPT1	5.96E-01	4.04E-04	postive
NDUFB10	5.96E-01	4.05E-04	postive
ALG6	5.96E-01	4.06E-04	postive
ECOP	5.96E-01	4.06E-04	postive
TIPRL	5.96E-01	4.08E-04	postive
MRPL15	5.96E-01	4.08E-04	postive
LOC441896	5.95E-01	4.10E-04	postive
RFFL	5.95E-01	4.11E-04	postive

MTERFD3	5.95E-01	4.11E-04	postive
TMEM185B	5.95E-01	4.12E-04	postive
USP46	5.95E-01	4.17E-04	postive
SAMD7	5.95E-01	4.17E-04	postive
DCUN1D4	5.95E-01	4.17E-04	postive
EEF1A1	5.95E-01	4.18E-04	postive
TMEM9B	5.95E-01	4.19E-04	postive
KIAA0232	5.95E-01	4.19E-04	postive
ATPIF1	5.95E-01	4.19E-04	postive
PIK3C3	5.95E-01	4.19E-04	postive
FN3KRP	5.94E-01	4.21E-04	postive
UXT	5.94E-01	4.22E-04	postive
NDUFS3	5.94E-01	4.23E-04	postive
COL4A1	-5.94E-01	4.23E-04	negative
FAM168B	5.94E-01	4.24E-04	postive
SNAPC5	5.94E-01	4.26E-04	postive
MGRN1	-5.94E-01	4.27E-04	negative
VTA1	5.94E-01	4.28E-04	postive
FAM60A	-5.94E-01	4.28E-04	negative
SPIN3	5.94E-01	4.29E-04	postive
HOMER1	5.94E-01	4.31E-04	postive
PLVAP	-5.94E-01	4.32E-04	negative
HNRNPA1	5.93E-01	4.33E-04	postive
GTF2A2	5.93E-01	4.34E-04	postive
COX8A	5.93E-01	4.34E-04	postive
PCMTD2	5.93E-01	4.34E-04	postive
ZNF622	5.93E-01	4.35E-04	postive
COG3	5.93E-01	4.36E-04	postive
IFI16	-5.93E-01	4.38E-04	negative
GPS1	5.93E-01	4.42E-04	postive
LGR4	5.93E-01	4.42E-04	postive
TXNDC17	5.93E-01	4.42E-04	postive
DYNC1I2	5.93E-01	4.43E-04	postive
LCLAT1	5.92E-01	4.45E-04	postive
EXOC3L2	-5.92E-01	4.46E-04	negative
GLUL	5.92E-01	4.50E-04	postive
VAPA	5.92E-01	4.51E-04	postive
CAPN2	5.92E-01	4.52E-04	postive
NUPR1	-5.92E-01	4.52E-04	negative
INO80E	-5.92E-01	4.54E-04	negative
PIGM	5.91E-01	4.60E-04	postive
ZFR	5.91E-01	4.62E-04	postive
DUSP8	-5.91E-01	4.64E-04	negative
COQ5	5.91E-01	4.64E-04	postive

SEC22C	5.91E-01	4.65E-04	postive
C18orf8	5.91E-01	4.66E-04	postive
CDC40	5.91E-01	4.68E-04	postive
PPP3CB	5.90E-01	4.71E-04	postive
MYL6	5.90E-01	4.72E-04	postive
SRI	5.90E-01	4.73E-04	postive
C12orf35	5.90E-01	4.74E-04	postive
C14orf126	5.90E-01	4.76E-04	postive
SIP1	5.90E-01	4.77E-04	postive
RELB	-5.90E-01	4.77E-04	negative
NUS1	5.90E-01	4.81E-04	postive
PPP3CA	5.90E-01	4.81E-04	postive
ELOVL4	5.89E-01	4.85E-04	postive
PDXDC1	5.89E-01	4.86E-04	postive
PDLIM5	-5.89E-01	4.87E-04	negative
CHP	5.89E-01	4.88E-04	postive
POMP	5.89E-01	4.88E-04	postive
LOC388122	5.89E-01	4.93E-04	postive
KRT18P42	-5.89E-01	4.93E-04	negative
GEMIN6	5.89E-01	4.95E-04	postive
MAP9	5.89E-01	4.95E-04	postive
WASF2	-5.89E-01	4.97E-04	negative
MESDC2	5.88E-01	4.97E-04	postive
STRC	-5.88E-01	4.99E-04	negative
FBXO9	5.88E-01	5.03E-04	postive
CARKD	5.88E-01	5.09E-04	postive
TAF7	5.88E-01	5.10E-04	postive
PFKFB2	5.88E-01	5.11E-04	postive
PSMA4	5.88E-01	5.11E-04	postive
CCDC5	5.87E-01	5.14E-04	postive
DYNC2LI1	5.87E-01	5.14E-04	postive
FAM134B	5.87E-01	5.18E-04	postive
CALY	5.87E-01	5.19E-04	postive
ARFGAP3	5.87E-01	5.19E-04	postive
LOC728449	-5.87E-01	5.20E-04	negative
C1orf168	-5.87E-01	5.23E-04	negative
UBE2O	-5.87E-01	5.24E-04	negative
MRPL4	5.87E-01	5.25E-04	postive
TRIO	-5.86E-01	5.26E-04	negative
LOC728732	5.86E-01	5.28E-04	postive
ATG5	5.86E-01	5.28E-04	postive
MTMR15	5.86E-01	5.31E-04	postive
SRP9	5.86E-01	5.31E-04	postive
KRT18P30	-5.86E-01	5.32E-04	negative

TCEAL1	5.86E-01	5.33E-04	postive
UQCRFS1	5.86E-01	5.34E-04	postive
LARP7	5.86E-01	5.36E-04	postive
ATP9B	5.86E-01	5.39E-04	postive
HMGCS1	5.85E-01	5.41E-04	postive
NAV1	-5.85E-01	5.44E-04	negative
PEX19	5.85E-01	5.45E-04	postive
CTSG	-5.85E-01	5.49E-04	negative
FOXN3	-5.85E-01	5.51E-04	negative
DMXL1	5.85E-01	5.51E-04	postive
PSMB5	5.85E-01	5.51E-04	postive
DHX29	5.85E-01	5.54E-04	postive
SERGEF	-5.84E-01	5.55E-04	negative
HSPA6	-5.84E-01	5.56E-04	negative
NAPEPLD	5.84E-01	5.58E-04	postive
LOC441073	5.84E-01	5.61E-04	postive
SLITRK5	5.84E-01	5.64E-04	postive
COL1A1	-5.84E-01	5.66E-04	negative
MORF4L2	5.84E-01	5.68E-04	postive
C1orf107	5.84E-01	5.69E-04	postive
VDAC1	5.84E-01	5.69E-04	postive
CASP4	-5.83E-01	5.71E-04	negative
LOC642076	5.83E-01	5.74E-04	postive
PPA1	5.83E-01	5.75E-04	postive
KPNA5	5.83E-01	5.76E-04	postive
THYN1	5.83E-01	5.77E-04	postive
CLIP1	5.83E-01	5.77E-04	postive
LOC100190890	-5.83E-01	5.77E-04	negative
SFRS2B	5.83E-01	5.78E-04	postive
HMGCR	5.83E-01	5.79E-04	postive
PAWR	-5.83E-01	5.79E-04	negative
DIO3OS	-5.83E-01	5.80E-04	negative
SAFB2	-5.83E-01	5.81E-04	negative
ANGPTL2	-5.83E-01	5.82E-04	negative
XRCC4	5.83E-01	5.82E-04	postive
SNX15	5.83E-01	5.82E-04	postive
DKFZP586B0319	-5.83E-01	5.83E-04	negative
GDF15	-5.83E-01	5.84E-04	negative
NT5C3	5.83E-01	5.84E-04	postive
RWDD1	5.83E-01	5.84E-04	postive
DZIP3	5.83E-01	5.85E-04	postive
CRYZ	5.82E-01	5.88E-04	postive
PXMP2	5.82E-01	5.89E-04	postive
C10orf35	5.82E-01	5.89E-04	postive

GTF3A	5.82E-01	5.89E-04	postive
C1orf31	5.82E-01	5.91E-04	postive
LOC728820	-5.82E-01	5.95E-04	negative
RTN4	5.82E-01	5.95E-04	postive
MTHFS	5.82E-01	5.95E-04	postive
S100A12	-5.82E-01	5.96E-04	negative
TEAD4	-5.82E-01	5.97E-04	negative
URB2	5.82E-01	6.00E-04	postive
BATF	-5.82E-01	6.00E-04	negative
PPP2CA	5.82E-01	6.00E-04	postive
QTRTD1	5.82E-01	6.01E-04	postive
POLR1C	5.81E-01	6.02E-04	postive
DAGLB	5.81E-01	6.02E-04	postive
VPS26A	5.81E-01	6.05E-04	postive
GPRASP1	5.81E-01	6.07E-04	postive
STAT5A	-5.81E-01	6.08E-04	negative
FCER1G	-5.81E-01	6.09E-04	negative
THTPA	5.81E-01	6.09E-04	postive
CCDC117	5.81E-01	6.09E-04	postive
SLC25A11	5.81E-01	6.11E-04	postive
SDS	-5.81E-01	6.12E-04	negative
KIAA0196	5.80E-01	6.19E-04	postive
FLJ10213	-5.80E-01	6.22E-04	negative
C4orf39	5.80E-01	6.28E-04	postive
POT1	5.80E-01	6.29E-04	postive
C13orf37	5.80E-01	6.30E-04	postive
CCDC86	-5.80E-01	6.30E-04	negative
C10orf141	5.80E-01	6.30E-04	postive
LOC643932	5.80E-01	6.32E-04	postive
TCEB1	5.80E-01	6.34E-04	postive
TMED5	5.79E-01	6.37E-04	postive
TM9SF3	5.79E-01	6.39E-04	postive
DPH2	5.79E-01	6.40E-04	postive
SLC2A14	-5.79E-01	6.45E-04	negative
SYNE1	5.79E-01	6.45E-04	postive
OPA1	5.79E-01	6.47E-04	postive
CBX3	5.79E-01	6.47E-04	postive
CYP1B1	-5.79E-01	6.49E-04	negative
HSPA8	5.79E-01	6.50E-04	postive
WWTR1	-5.79E-01	6.50E-04	negative
KIAA0090	5.79E-01	6.51E-04	postive
MYCT1	-5.78E-01	6.53E-04	negative
JUND	-5.78E-01	6.54E-04	negative
ATP13A3	5.78E-01	6.54E-04	postive

AEBP1	-5.78E-01	6.58E-04	negative
C9orf80	5.78E-01	6.59E-04	postive
TNFRSF1A	-5.78E-01	6.60E-04	negative
PSMB2	5.78E-01	6.60E-04	postive
NR3C2	5.78E-01	6.61E-04	postive
EIF3I	5.78E-01	6.62E-04	postive
TUSC2	5.78E-01	6.64E-04	postive
DUSP10	5.78E-01	6.65E-04	postive
AAMP	5.78E-01	6.65E-04	postive
TFAM	5.78E-01	6.68E-04	postive
C10orf10	-5.78E-01	6.68E-04	negative
ICAM1	-5.77E-01	6.75E-04	negative
MMP2	-5.77E-01	6.76E-04	negative
GMPS	-5.77E-01	6.76E-04	negative
PTGES3	5.77E-01	6.78E-04	postive
UCHL5	5.77E-01	6.78E-04	postive
TNKS	5.77E-01	6.79E-04	postive
PPP1R3E	-5.77E-01	6.81E-04	negative
MADD	5.77E-01	6.82E-04	postive
ISG20	-5.77E-01	6.82E-04	negative
C1orf96	-5.77E-01	6.82E-04	negative
WDR77	5.77E-01	6.85E-04	postive
MCM3AP	5.77E-01	6.86E-04	postive
RHBDD2	5.77E-01	6.87E-04	postive
HIST2H2BE	5.76E-01	6.89E-04	postive
STRADB	5.76E-01	6.91E-04	postive
SLC11A1	-5.76E-01	6.94E-04	negative
EEF1D	-5.76E-01	6.95E-04	negative
BBX	-5.76E-01	6.96E-04	negative
TFEB	-5.76E-01	6.96E-04	negative
NPM1	5.76E-01	6.98E-04	postive
JMJD2D	5.76E-01	6.98E-04	postive
IGHMBP2	5.76E-01	7.01E-04	postive
C1orf38	-5.76E-01	7.02E-04	negative
SKP1	5.76E-01	7.02E-04	postive
SENP2	5.76E-01	7.05E-04	postive
EIF2S2	5.76E-01	7.06E-04	postive
GNE	5.75E-01	7.07E-04	postive
INPP5E	5.75E-01	7.11E-04	postive
POLH	-5.75E-01	7.12E-04	negative
C14orf135	5.75E-01	7.12E-04	postive
UBTF	5.75E-01	7.13E-04	postive
UBR1	5.75E-01	7.16E-04	postive
HAX1	5.75E-01	7.17E-04	postive

SFRS18	-5.75E-01	7.18E-04	negative
TMEM147	5.75E-01	7.18E-04	postive
ZNF347	5.75E-01	7.18E-04	postive
LOC401397	5.75E-01	7.20E-04	postive
UBAP2	5.75E-01	7.20E-04	postive
DCTN6	5.75E-01	7.21E-04	postive
C8orf58	-5.75E-01	7.22E-04	negative
C17orf42	5.75E-01	7.23E-04	postive
IL10RA	-5.75E-01	7.24E-04	negative
MRS2	5.75E-01	7.24E-04	postive
CD93	-5.74E-01	7.25E-04	negative
LYPLA2P1	5.74E-01	7.27E-04	postive
NCOA7	5.74E-01	7.29E-04	postive
EPS8L2	-5.74E-01	7.29E-04	negative
FAM21A	5.74E-01	7.29E-04	postive
KIAA1545	-5.74E-01	7.29E-04	negative
CCDC90B	5.74E-01	7.30E-04	postive
RPL18	5.74E-01	7.33E-04	postive
SRP14	5.74E-01	7.34E-04	postive
STOM	-5.74E-01	7.36E-04	negative
CLDN3	-5.74E-01	7.38E-04	negative
CST7	-5.74E-01	7.42E-04	negative
TRRAP	5.74E-01	7.43E-04	postive
GJA4	-5.73E-01	7.45E-04	negative
METTL2B	5.73E-01	7.47E-04	postive
PRR17	-5.73E-01	7.48E-04	negative
HNRNPA1L2	5.73E-01	7.48E-04	postive
AQP1	-5.73E-01	7.49E-04	negative
RWDD3	5.73E-01	7.52E-04	postive
DACT1	-5.73E-01	7.59E-04	negative
ZNF30	5.73E-01	7.63E-04	postive
WDR61	5.72E-01	7.65E-04	postive
COX7A2L	5.72E-01	7.65E-04	postive
VEZT	5.72E-01	7.65E-04	postive
RDH12	5.72E-01	7.67E-04	postive
SLCO4A1	-5.72E-01	7.70E-04	negative
H19	-5.72E-01	7.70E-04	negative
SETD6	5.72E-01	7.71E-04	postive
MBTD1	5.72E-01	7.72E-04	postive
SGTA	-5.72E-01	7.74E-04	negative
NDFIP2	5.72E-01	7.74E-04	postive
CACNA2D3	5.72E-01	7.74E-04	postive
IL3RA	-5.72E-01	7.75E-04	negative
CLASP2	5.72E-01	7.77E-04	postive

USP8	5.72E-01	7.80E-04	postive
RRN3	5.72E-01	7.82E-04	postive
MDH2	5.72E-01	7.83E-04	postive
ZNF135	5.72E-01	7.84E-04	postive
CCDC44	5.71E-01	7.85E-04	postive
IMPAD1	5.71E-01	7.86E-04	postive
SH3GL3	5.71E-01	7.87E-04	postive
KBTBD7	5.71E-01	7.88E-04	postive
ZNF271	5.71E-01	7.89E-04	postive
APOL1	-5.71E-01	7.93E-04	negative
LOC26010	5.71E-01	7.93E-04	postive
SRPK2	5.71E-01	7.94E-04	postive
SLC30A9	5.71E-01	7.95E-04	postive
S100P	-5.71E-01	8.00E-04	negative
ERGIC1	-5.71E-01	8.01E-04	negative
CNDP1	-5.70E-01	8.07E-04	negative
TTC33	5.70E-01	8.07E-04	postive
POLR2H	-5.70E-01	8.08E-04	negative
TTRAP	5.70E-01	8.14E-04	postive
LCE1D	-5.70E-01	8.19E-04	negative
TCF7L1	-5.70E-01	8.19E-04	negative
MDM1	5.70E-01	8.20E-04	postive
NTHL1	5.70E-01	8.25E-04	postive
HADH	5.69E-01	8.28E-04	postive
KRCC1	5.69E-01	8.29E-04	postive
FUT8	5.69E-01	8.32E-04	postive
C16orf88	5.69E-01	8.33E-04	postive
SCAND3	5.69E-01	8.33E-04	postive
LRCH3	5.69E-01	8.34E-04	postive
AP2A2	-5.69E-01	8.41E-04	negative
NUP88	5.68E-01	8.50E-04	postive
SPAG9	5.68E-01	8.50E-04	postive
CREBL2	5.68E-01	8.51E-04	postive
TGFB1	-5.68E-01	8.53E-04	negative
ABCD4	5.68E-01	8.56E-04	postive
TEX261	5.68E-01	8.61E-04	postive
S100A9	-5.68E-01	8.61E-04	negative
PSMD5	5.68E-01	8.64E-04	postive
TGOLN2	5.68E-01	8.65E-04	postive
SAE1	5.67E-01	8.79E-04	postive
EEF1B2	5.67E-01	8.80E-04	postive
RNF34	5.67E-01	8.85E-04	postive
ZNRD1	5.67E-01	8.86E-04	postive
ZNF546	5.67E-01	8.87E-04	postive

SOCS3	-5.67E-01	8.89E-04	negative
SEMA3A	5.66E-01	8.97E-04	postive
COX7A2	5.66E-01	9.00E-04	postive
CALM1	5.66E-01	9.02E-04	postive
GGCT	5.66E-01	9.03E-04	postive
CHAC2	5.66E-01	9.07E-04	postive
CRYZL1	5.66E-01	9.11E-04	postive
TRAM1L1	5.66E-01	9.11E-04	postive
SCARB1	-5.66E-01	9.12E-04	negative
PTS	5.66E-01	9.14E-04	postive
YARS2	5.65E-01	9.17E-04	postive
GTF2H5	5.65E-01	9.23E-04	postive
LOC100131601	-5.65E-01	9.24E-04	negative
SPON1	5.65E-01	9.24E-04	postive
ATP5G1	5.65E-01	9.25E-04	postive
CMKLR1	-5.65E-01	9.26E-04	negative
EEF2	5.65E-01	9.30E-04	postive
KIAA1370	5.65E-01	9.30E-04	postive
SNAP25	5.65E-01	9.34E-04	postive
AQP9	-5.65E-01	9.37E-04	negative
S1PR4	-5.64E-01	9.44E-04	negative
C1orf103	5.64E-01	9.46E-04	postive
HBM	-5.64E-01	9.51E-04	negative
NBR1	5.64E-01	9.52E-04	postive
CD8A	-5.64E-01	9.56E-04	negative
PTPLAD1	5.63E-01	9.64E-04	postive
RAPGEF2	5.63E-01	9.70E-04	postive
CUL4B	5.63E-01	9.73E-04	postive
PRO0478	-5.63E-01	9.77E-04	negative
HEG1	5.63E-01	9.78E-04	postive
MAPK10	5.63E-01	9.81E-04	postive
C15orf23	5.63E-01	9.81E-04	postive
ZNF554	5.63E-01	9.85E-04	postive
LOC440895	5.63E-01	9.88E-04	postive
TMEM60	5.62E-01	9.89E-04	postive
PGRMC2	-5.62E-01	9.90E-04	negative
ORAI1	-5.62E-01	9.98E-04	negative
TASP1	5.62E-01	9.98E-04	postive
C3orf23	5.62E-01	9.98E-04	postive
FLJ40142	7.53E-01	1.00E-06	postive
TSKU	-7.53E-01	1.00E-06	negative
CLDND1	7.03E-01	1.01E-05	postive
NOL7	7.53E-01	1.02E-06	postive
FLJ10038	7.03E-01	1.04E-05	postive

SNRPC	7.53E-01	1.04E-06	postive
SFRS10	7.93E-01	1.06E-07	postive
BRWD2	7.02E-01	1.07E-05	postive
TM9SF2	7.52E-01	1.08E-06	postive
CNBP	7.52E-01	1.09E-06	postive
ARPC1A	7.92E-01	1.09E-07	postive
XPOT	7.02E-01	1.10E-05	postive
HSBP1	7.01E-01	1.11E-05	postive
PDC	7.01E-01	1.12E-05	postive
FBXO3	7.51E-01	1.12E-06	postive
ACOT2	7.01E-01	1.13E-05	postive
ZADH2	-7.01E-01	1.14E-05	negative
ZNF444	-7.01E-01	1.14E-05	negative
CISD1	7.00E-01	1.15E-05	postive
PCNP	7.00E-01	1.15E-05	postive
RBBP6	-7.51E-01	1.15E-06	negative
PSMA1	7.91E-01	1.15E-07	postive
BDKRB2	-7.00E-01	1.16E-05	negative
SEC14L1	-7.00E-01	1.16E-05	negative
TXNDC13	7.00E-01	1.16E-05	postive
TBCC	7.00E-01	1.17E-05	postive
ATXN10	8.25E-01	1.17E-08	postive
C1QBP	7.50E-01	1.18E-06	postive
CCT6A	8.52E-01	1.18E-09	postive
PSMA6	7.00E-01	1.19E-05	postive
EEF1E1	6.99E-01	1.20E-05	postive
FLJ23185	-6.99E-01	1.21E-05	negative
HTRA2	6.99E-01	1.22E-05	postive
CXXC5	-7.90E-01	1.25E-07	negative
DDX47	7.49E-01	1.26E-06	postive
HSPA9	7.90E-01	1.27E-07	postive
KIAA1147	6.98E-01	1.28E-05	postive
TAF1B	6.98E-01	1.28E-05	postive
FAM50B	7.49E-01	1.28E-06	postive
NDUFB6	7.90E-01	1.28E-07	postive
SCFD1	7.48E-01	1.29E-06	postive
POLR2B	6.97E-01	1.30E-05	postive
GFM1	7.89E-01	1.30E-07	postive
ATXN7	-6.97E-01	1.31E-05	negative
MAP3K3	-6.97E-01	1.32E-05	negative
COPS5	7.48E-01	1.32E-06	postive
C21orf33	6.97E-01	1.33E-05	postive
RCN2	6.97E-01	1.34E-05	postive
SRPRB	7.48E-01	1.34E-06	postive

TMEM50B	7.89E-01	1.34E-07	postive
OSBPL11	6.97E-01	1.35E-05	postive
SEC11C	6.97E-01	1.35E-05	postive
ECHS1	7.47E-01	1.36E-06	postive
FUSIP1	6.96E-01	1.37E-05	postive
BRP44L	6.96E-01	1.39E-05	postive
STARD7	6.96E-01	1.39E-05	postive
UCHL3	6.96E-01	1.39E-05	postive
SERINC1	6.95E-01	1.41E-05	postive
MRPL3	8.50E-01	1.41E-09	postive
TRIM44	6.95E-01	1.45E-05	postive
SGEF	6.94E-01	1.47E-05	postive
PRPSAP1	7.87E-01	1.47E-07	postive
USP1	7.46E-01	1.48E-06	postive
XPA	7.46E-01	1.48E-06	postive
POLR2G	6.94E-01	1.49E-05	postive
GPI	-7.46E-01	1.49E-06	negative
ARMCX2	7.45E-01	1.50E-06	postive
CNIH	6.94E-01	1.52E-05	postive
STAC2	-6.93E-01	1.52E-05	negative
TMEM203	6.93E-01	1.53E-05	postive
OCIAD1	7.87E-01	1.53E-07	postive
MRPL9	7.45E-01	1.54E-06	postive
MAPRE1	6.93E-01	1.57E-05	postive
RSL1D1	7.44E-01	1.57E-06	postive
RASSF7	-7.86E-01	1.58E-07	negative
ACTR6	6.92E-01	1.59E-05	postive
PPP2CB	7.44E-01	1.59E-06	postive
JMJD1B	6.92E-01	1.60E-05	postive
ZKSCAN1	-6.92E-01	1.61E-05	negative
GPAM	6.92E-01	1.62E-05	postive
MRPS31	6.92E-01	1.62E-05	postive
RAB1A	6.92E-01	1.63E-05	postive
SMC3	6.92E-01	1.63E-05	postive
TMEM14B	6.92E-01	1.63E-05	postive
BUD31	6.91E-01	1.66E-05	postive
ZBTB7A	-6.91E-01	1.66E-05	negative
PSMD14	8.20E-01	1.67E-08	postive
MNT	-7.43E-01	1.68E-06	negative
DHX36	7.85E-01	1.70E-07	postive
ARRDC2	-8.20E-01	1.70E-08	negative
GMFB	8.20E-01	1.71E-08	postive
CRADD	6.90E-01	1.74E-05	postive
DSTN	6.90E-01	1.74E-05	postive

TSN	6.90E-01	1.76E-05	postive
FXC1	6.90E-01	1.77E-05	postive
NME1	7.84E-01	1.79E-07	postive
PELI2	7.84E-01	1.80E-07	postive
MAGEH1	8.48E-01	1.80E-09	postive
C3orf14	6.89E-01	1.81E-05	postive
UBA2	6.89E-01	1.81E-05	postive
LRRC49	7.42E-01	1.81E-06	postive
TPD52	6.89E-01	1.83E-05	postive
F11R	-6.89E-01	1.84E-05	negative
ARFGEF1	6.89E-01	1.85E-05	postive
NOL3	-6.88E-01	1.86E-05	negative
B3GALT6	7.84E-01	1.86E-07	postive
EHMT1	6.88E-01	1.87E-05	postive
PSMB4	6.88E-01	1.87E-05	postive
SBDS	6.88E-01	1.87E-05	postive
TRAF3IP1	7.83E-01	1.87E-07	postive
GFM2	8.18E-01	1.87E-08	postive
NUP160	7.41E-01	1.88E-06	postive
SEC23B	7.40E-01	1.92E-06	postive
SCYL3	6.87E-01	1.94E-05	postive
UBR2	6.87E-01	1.94E-05	postive
KIAA0317	8.18E-01	1.94E-08	postive
NARS	7.40E-01	1.95E-06	postive
SLC25A17	6.87E-01	1.96E-05	postive
C15orf24	6.87E-01	1.97E-05	postive
PPP2R3C	7.83E-01	1.97E-07	postive
CACNB2	6.87E-01	1.98E-05	postive
HERC1	6.87E-01	2.00E-05	postive
ADD3	6.86E-01	2.01E-05	postive
APPBP2	6.86E-01	2.02E-05	postive
PTBP2	6.86E-01	2.02E-05	postive
TRMT12	6.86E-01	2.02E-05	postive
C15orf28	-6.86E-01	2.04E-05	negative
CUL4A	6.86E-01	2.04E-05	postive
LDOC1L	6.86E-01	2.04E-05	postive
SLC25A33	6.86E-01	2.04E-05	postive
FOXD2	-7.82E-01	2.04E-07	negative
NME5	6.86E-01	2.07E-05	postive
UBE2A	7.39E-01	2.09E-06	postive
PTPRF	-6.85E-01	2.10E-05	negative
CNGA1	6.85E-01	2.11E-05	postive
PHLPPL	6.85E-01	2.11E-05	postive
ZNF395	-6.85E-01	2.11E-05	negative

PPIL1	6.85E-01	2.13E-05	postive
DIABLO	7.38E-01	2.13E-06	postive
MLH1	6.85E-01	2.15E-05	postive
MED4	7.38E-01	2.18E-06	postive
PLRG1	7.38E-01	2.20E-06	postive
ZNF655	8.16E-01	2.21E-08	postive
MFAP1	7.37E-01	2.22E-06	postive
BICD2	8.69E-01	2.23E-10	postive
BCL7B	6.84E-01	2.24E-05	postive
TMCO1	6.84E-01	2.25E-05	postive
EXOC1	7.37E-01	2.25E-06	postive
PSMD9	7.37E-01	2.25E-06	postive
TSPYL1	7.37E-01	2.25E-06	postive
NAP1L5	6.83E-01	2.26E-05	postive
WDSOF1	7.37E-01	2.26E-06	postive
SH3BGRL3	-6.83E-01	2.32E-05	negative
RPS6KB1	7.37E-01	2.32E-06	postive
GLRX3	7.80E-01	2.32E-07	postive
TXNDC9	6.83E-01	2.33E-05	postive
EIF3J	6.82E-01	2.37E-05	postive
SPHK1	-6.82E-01	2.37E-05	negative
HSD11B1	-7.36E-01	2.39E-06	negative
GTF3C6	7.79E-01	2.39E-07	postive
SLC35A1	7.36E-01	2.40E-06	postive
ATP5A1	6.82E-01	2.41E-05	postive
RARA	-7.79E-01	2.41E-07	negative
PDZD7	-6.82E-01	2.42E-05	negative
WIPF1	-6.82E-01	2.42E-05	negative
OAT	7.36E-01	2.42E-06	postive
RTN4IP1	6.81E-01	2.43E-05	postive
MRPS35	7.35E-01	2.44E-06	postive
ZNF75D	8.15E-01	2.44E-08	postive
B4GALT1	-6.81E-01	2.49E-05	negative
CD40	-6.81E-01	2.49E-05	negative
WDR3	6.81E-01	2.49E-05	postive
NFS1	6.81E-01	2.50E-05	postive
MAPKAPK5	6.80E-01	2.54E-05	postive
SAP18	8.68E-01	2.54E-10	postive
VEGFA	-6.80E-01	2.55E-05	negative
ZFP112	6.80E-01	2.57E-05	postive
C10orf32	7.78E-01	2.57E-07	postive
SF3B14	6.80E-01	2.60E-05	postive
SH3GL2	6.79E-01	2.63E-05	postive
TAF9	6.79E-01	2.63E-05	postive

TMTC4	6.80E-01	2.63E-05	postive
MAN2B1	-6.79E-01	2.65E-05	negative
NUP133	6.79E-01	2.65E-05	postive
PMS1	6.79E-01	2.65E-05	postive
RRAGC	-6.79E-01	2.65E-05	negative
GPR125	7.77E-01	2.72E-07	postive
GPM6A	6.79E-01	2.73E-05	postive
MRPL16	7.33E-01	2.73E-06	postive
MRLC2	8.13E-01	2.73E-08	postive
FMN1	6.78E-01	2.74E-05	postive
SCRN3	7.33E-01	2.74E-06	postive
NHS	7.77E-01	2.74E-07	postive
SLC25A5	6.78E-01	2.75E-05	postive
HAT1	7.33E-01	2.75E-06	postive
PSMF1	7.33E-01	2.75E-06	postive
TDRD7	7.33E-01	2.75E-06	postive
RAD17	7.77E-01	2.76E-07	postive
IDE	7.77E-01	2.79E-07	postive
CD200	6.78E-01	2.82E-05	postive
ACSL6	7.32E-01	2.84E-06	postive
ZDHHC13	6.77E-01	2.85E-05	postive
MREG	7.32E-01	2.85E-06	postive
NHP2L1	6.77E-01	2.91E-05	postive
CCT6P1	7.32E-01	2.92E-06	postive
CASP8AP2	6.77E-01	2.93E-05	postive
PLEKHO1	-6.76E-01	2.95E-05	negative
FAM82A2	7.31E-01	2.95E-06	postive
UBE2E3	7.76E-01	2.95E-07	postive
EED	6.76E-01	2.98E-05	postive
LEPROTL1	8.12E-01	2.98E-08	postive
FBXO8	7.31E-01	3.00E-06	postive
C7orf28B	7.75E-01	3.00E-07	postive
CHCHD4	6.76E-01	3.01E-05	postive
PJA1	7.31E-01	3.03E-06	postive
RFX5	6.76E-01	3.05E-05	postive
ENOPH1	6.75E-01	3.06E-05	postive
RASL12	-6.75E-01	3.07E-05	negative
BHLHB9	7.30E-01	3.09E-06	postive
ADSS	6.75E-01	3.10E-05	postive
CASC4	6.75E-01	3.12E-05	postive
ATP5F1	6.75E-01	3.14E-05	postive
LSP1	-6.75E-01	3.14E-05	negative
PWP1	6.75E-01	3.14E-05	postive
MDH1	6.74E-01	3.19E-05	postive

PSMB7	6.74E-01	3.19E-05	postive
FANCL	7.74E-01	3.19E-07	postive
DACH1	7.29E-01	3.26E-06	postive
C5orf44	6.73E-01	3.29E-05	postive
KIAA2018	6.73E-01	3.29E-05	postive
ZNF180	6.73E-01	3.31E-05	postive
DLD	7.29E-01	3.32E-06	postive
PRMT6	6.73E-01	3.39E-05	postive
TIMM17A	7.73E-01	3.39E-07	postive
FLJ22795	-6.73E-01	3.40E-05	negative
DHX9	6.72E-01	3.41E-05	postive
MTX2	7.28E-01	3.41E-06	postive
COPS4	8.10E-01	3.41E-08	postive
LOC390424	7.28E-01	3.42E-06	postive
RAD23B	6.72E-01	3.43E-05	postive
GPR4	-6.72E-01	3.45E-05	negative
TJP1	6.72E-01	3.47E-05	postive
PNRC2	7.28E-01	3.48E-06	postive
MRPL24	6.72E-01	3.49E-05	postive
EIF4G2	6.72E-01	3.53E-05	postive
SLC38A1	6.71E-01	3.54E-05	postive
SEC61B	6.71E-01	3.55E-05	postive
TCAP	-6.71E-01	3.55E-05	negative
SELS	7.72E-01	3.56E-07	postive
TPRKB	6.71E-01	3.57E-05	postive
DCTPP1	7.27E-01	3.59E-06	postive
ACAT1	7.27E-01	3.61E-06	postive
C16orf63	7.72E-01	3.61E-07	postive
UBE2N	7.27E-01	3.62E-06	postive
GNG10	7.27E-01	3.63E-06	postive
MIA3	7.26E-01	3.74E-06	postive
FCHSD2	6.70E-01	3.75E-05	postive
ERH	8.08E-01	3.78E-08	postive
SC5DL	6.70E-01	3.79E-05	postive
ALS2CR4	6.69E-01	3.83E-05	postive
SEC23A	6.69E-01	3.83E-05	postive
TMEM17	6.69E-01	3.85E-05	postive
SUCLA2	6.69E-01	3.86E-05	postive
B3GALNT1	7.26E-01	3.86E-06	postive
CEBPE	-6.69E-01	3.87E-05	negative
DNAJA3	6.69E-01	3.87E-05	postive
BSDC1	-6.69E-01	3.88E-05	negative
SCO1	8.08E-01	3.88E-08	postive
CYTIP	-6.69E-01	3.90E-05	negative

MRPS27	6.69E-01	3.93E-05	postive
DOLK	6.68E-01	3.97E-05	postive
ACTR3B	7.25E-01	3.97E-06	postive
CD47	7.25E-01	3.98E-06	postive
C20orf142	7.70E-01	4.03E-07	postive
HINT1	6.68E-01	4.06E-05	postive
C15orf61	7.70E-01	4.06E-07	postive
PXN	-8.07E-01	4.09E-08	negative
HTATSF1	6.67E-01	4.12E-05	postive
PDCD10	6.67E-01	4.13E-05	postive
PGM2	6.67E-01	4.13E-05	postive
CISH	-7.24E-01	4.16E-06	negative
LRRC25	-6.67E-01	4.17E-05	negative
ORMDL2	6.67E-01	4.17E-05	postive
FBXL5	8.84E-01	4.20E-11	postive
AGPAT2	-6.67E-01	4.22E-05	negative
C20orf24	7.24E-01	4.23E-06	postive
VPS29	6.66E-01	4.26E-05	postive
TM2D2	7.23E-01	4.26E-06	postive
CBL	-6.66E-01	4.27E-05	negative
BBS7	6.66E-01	4.28E-05	postive
C21orf119	6.66E-01	4.28E-05	postive
KIAA1219	8.63E-01	4.30E-10	postive
GOLGA5	6.66E-01	4.37E-05	postive
SDR39U1	7.69E-01	4.40E-07	postive
HIGD1A	6.65E-01	4.43E-05	postive
GOPC	7.22E-01	4.52E-06	postive
IMPA1	8.37E-01	4.53E-09	postive
MRPS28	7.22E-01	4.54E-06	postive
ZNF562	6.64E-01	4.57E-05	postive
ITPA	6.64E-01	4.60E-05	postive
BTRC	6.64E-01	4.67E-05	postive
BEX1	8.05E-01	4.68E-08	postive
COMMD1	6.64E-01	4.70E-05	postive
RBX1	8.05E-01	4.70E-08	postive
NSD1	6.63E-01	4.75E-05	postive
CYB5R1	7.21E-01	4.76E-06	postive
KLHL9	7.67E-01	4.79E-07	postive
ZBTB16	-6.63E-01	4.83E-05	negative
C7orf36	6.63E-01	4.85E-05	postive
RCBTB1	7.20E-01	4.88E-06	postive
LENG8	-7.67E-01	4.89E-07	negative
NDUFC2	7.67E-01	4.89E-07	postive
PPP1R9B	-7.20E-01	4.90E-06	negative

POLR3F	7.20E-01	4.92E-06	postive
UBAC1	7.20E-01	4.93E-06	postive
SYVN1	6.62E-01	4.98E-05	postive
DDX5	6.62E-01	4.99E-05	postive
PRR11	-6.62E-01	5.02E-05	negative
C11orf67	-6.62E-01	5.06E-05	negative
FAM96A	7.66E-01	5.06E-07	postive
SRFBP1	6.61E-01	5.09E-05	postive
DPY19L4	6.61E-01	5.13E-05	postive
POLR3K	6.61E-01	5.13E-05	postive
RABEPK	7.66E-01	5.13E-07	postive
HARS2	6.61E-01	5.14E-05	postive
NKIRAS1	6.61E-01	5.14E-05	postive
CASP3	6.61E-01	5.16E-05	postive
SELT	6.61E-01	5.16E-05	postive
C20orf30	6.61E-01	5.17E-05	postive
CCT5	6.61E-01	5.17E-05	postive
ZNF706	7.19E-01	5.17E-06	postive
SCHIP1	7.19E-01	5.19E-06	postive
CCDC53	6.61E-01	5.20E-05	postive
YWHAQ	6.61E-01	5.24E-05	postive
C9orf5	7.19E-01	5.28E-06	postive
CAMTA1	6.60E-01	5.30E-05	postive
MRPS33	6.60E-01	5.30E-05	postive
TAF11	6.60E-01	5.31E-05	postive
TSFM	6.60E-01	5.32E-05	postive
PAIP1	6.60E-01	5.34E-05	postive
NUDT9	6.60E-01	5.36E-05	postive
PDCD6	8.03E-01	5.37E-08	postive
ATP5J	7.65E-01	5.38E-07	postive
IARS	7.18E-01	5.39E-06	postive
SEC13	6.60E-01	5.41E-05	postive
C19orf50	-6.60E-01	5.42E-05	negative
GTPBP4	8.34E-01	5.42E-09	postive
MMP14	-7.18E-01	5.43E-06	negative
CETN2	7.65E-01	5.43E-07	postive
TFG	7.65E-01	5.47E-07	postive
NUP35	7.65E-01	5.50E-07	postive
TBC1D3	-6.59E-01	5.51E-05	negative
GCC1	6.59E-01	5.52E-05	postive
MRPL32	6.59E-01	5.54E-05	postive
TMEM126A	6.59E-01	5.56E-05	postive
POLR2C	7.17E-01	5.57E-06	postive
C14orf1	6.59E-01	5.58E-05	postive

DPH3	6.59E-01	5.59E-05	postive
EPRS	7.17E-01	5.62E-06	postive
HS3ST3B1	6.58E-01	5.65E-05	postive
PEX3	6.58E-01	5.69E-05	postive
RBL2	6.58E-01	5.73E-05	postive
SNAP47	6.58E-01	5.76E-05	postive
PXMP3	7.64E-01	5.79E-07	postive
NME2P1	6.58E-01	5.80E-05	postive
TMEM2	6.58E-01	5.80E-05	postive
PSMD6	7.64E-01	5.80E-07	postive
ATP6V1A	7.64E-01	5.83E-07	postive
PPT1	6.58E-01	5.84E-05	postive
TRUB1	6.58E-01	5.84E-05	postive
SF3A3	7.64E-01	5.84E-07	postive
C9orf167	-6.57E-01	5.86E-05	negative
HNRNPK	6.57E-01	5.86E-05	postive
PFKFB3	-6.57E-01	5.89E-05	negative
TTC7A	-6.57E-01	5.89E-05	negative
CCNDBP1	6.57E-01	5.90E-05	postive
SASH1	-6.57E-01	5.90E-05	negative
HACE1	6.57E-01	5.93E-05	postive
DDX1	7.63E-01	5.96E-07	postive
MPHOSPH6	7.63E-01	5.99E-07	postive
ZNF148	-7.63E-01	5.99E-07	negative
DNAJA2	7.63E-01	6.01E-07	postive
C17orf79	6.57E-01	6.02E-05	postive
FLJ45055	-7.16E-01	6.04E-06	negative
DMPK	-6.56E-01	6.10E-05	negative
NOL11	7.63E-01	6.10E-07	postive
SOX2	6.56E-01	6.14E-05	postive
FRY	7.15E-01	6.15E-06	postive
HBZ	-6.56E-01	6.24E-05	negative
PSMC6	6.56E-01	6.25E-05	postive
ARHGEF7	7.15E-01	6.26E-06	postive
IER3IP1	6.55E-01	6.32E-05	postive
ABI1	7.62E-01	6.32E-07	postive
LOC100190939	-6.55E-01	6.34E-05	negative
CAPN3	-6.55E-01	6.35E-05	negative
PSMB1	6.55E-01	6.35E-05	postive
IGFBP5	-6.55E-01	6.36E-05	negative
ADAD2	-6.55E-01	6.41E-05	negative
ACTR10	7.62E-01	6.42E-07	postive
HISPPD1	7.14E-01	6.47E-06	postive
MKI67IP	6.55E-01	6.48E-05	postive

CCDC56	8.00E-01	6.50E-08	postive
TTC27	6.54E-01	6.53E-05	postive
CHURC1	7.14E-01	6.55E-06	postive
ATP2A2	6.54E-01	6.56E-05	postive
ATP5G3	7.61E-01	6.57E-07	postive
GTF3C3	6.54E-01	6.60E-05	postive
DKFZp686E2433	7.13E-01	6.63E-06	postive
SDHB	6.54E-01	6.64E-05	postive
WDR35	6.54E-01	6.64E-05	postive
CDC123	7.13E-01	6.67E-06	postive
UPF3A	6.54E-01	6.69E-05	postive
MAP3K6	-6.54E-01	6.70E-05	negative
RBM18	6.54E-01	6.71E-05	postive
C1orf156	7.13E-01	6.73E-06	postive
LZIC	-6.53E-01	6.74E-05	negative
MGAT2	7.13E-01	6.75E-06	postive
COPS8	7.61E-01	6.76E-07	postive
CABIN1	-6.53E-01	6.81E-05	negative
ARID4B	6.53E-01	6.85E-05	postive
SNX2	7.13E-01	6.88E-06	postive
IFT57	7.12E-01	6.91E-06	postive
NAT10	6.53E-01	6.93E-05	postive
UQCC	6.53E-01	6.93E-05	postive
UBE4A	6.53E-01	6.94E-05	postive
OSTM1	7.60E-01	6.94E-07	postive
KIFAP3	6.52E-01	6.96E-05	postive
BEX2	7.60E-01	6.96E-07	postive
SLC4A1AP	7.60E-01	6.98E-07	postive
MSH2	7.12E-01	6.99E-06	postive
SLMO2	6.52E-01	7.02E-05	postive
DENND1A	-7.60E-01	7.02E-07	negative
OXCT1	7.60E-01	7.04E-07	postive
UNC50	7.60E-01	7.04E-07	postive
UBP1	7.12E-01	7.16E-06	postive
SFXN4	6.51E-01	7.23E-05	postive
SRRM2	-7.11E-01	7.23E-06	negative
C6orf130	7.60E-01	7.23E-07	postive
AGL	6.51E-01	7.26E-05	postive
GALNT11	7.59E-01	7.28E-07	postive
PIM1	-7.11E-01	7.31E-06	negative
TCEA1	7.11E-01	7.36E-06	postive
BCL3	-7.59E-01	7.37E-07	negative
SIDT2	-7.59E-01	7.39E-07	negative
LSM1	6.51E-01	7.41E-05	postive

GNL3	7.59E-01	7.44E-07	postive
C1orf162	-6.50E-01	7.47E-05	negative
CCDC17	-6.50E-01	7.47E-05	negative
CHCHD7	7.59E-01	7.50E-07	postive
RPL27	6.50E-01	7.51E-05	postive
VEGFB	-6.50E-01	7.51E-05	negative
TMEM42	7.10E-01	7.57E-06	postive
ZCCHC3	6.50E-01	7.62E-05	postive
ADAMTS7	-7.10E-01	7.64E-06	negative
PIGF	6.50E-01	7.66E-05	postive
HTRA3	-6.50E-01	7.68E-05	negative
JTB	7.58E-01	7.70E-07	postive
TMEM41B	7.10E-01	7.80E-06	postive
ENTPD4	7.58E-01	7.82E-07	postive
ATP2B1	7.09E-01	7.91E-06	postive
LOC728324	7.58E-01	7.96E-07	postive
BXDC5	6.48E-01	7.97E-05	postive
ZCCHC9	6.48E-01	7.97E-05	postive
CHMP2B	7.09E-01	7.97E-06	postive
MTMR9	8.56E-01	7.98E-10	postive
C14orf100	6.48E-01	8.00E-05	postive
HIVEP2	-6.48E-01	8.07E-05	negative
TSG101	7.58E-01	8.07E-07	postive
LIX1L	6.48E-01	8.14E-05	postive
NOP10	6.48E-01	8.14E-05	postive
NSMCE4A	6.48E-01	8.15E-05	postive
COPB2	7.09E-01	8.18E-06	postive
RAD51C	7.57E-01	8.19E-07	postive
DNHD1	-6.48E-01	8.21E-05	negative
PCSK2	6.48E-01	8.22E-05	postive
CMPK1	7.08E-01	8.23E-06	postive
NDUFAB1	7.97E-01	8.24E-08	postive
KCNQ1OT1	-7.08E-01	8.26E-06	negative
C1orf50	6.47E-01	8.28E-05	postive
UROS	6.47E-01	8.30E-05	postive
WDR12	6.47E-01	8.30E-05	postive
HSPE1	7.57E-01	8.30E-07	postive
TSPYL5	7.08E-01	8.31E-06	postive
PSMA5	7.08E-01	8.32E-06	postive
RTF1	6.47E-01	8.34E-05	postive
PDE4C	-6.47E-01	8.35E-05	negative
CDH3	-6.47E-01	8.41E-05	negative
CCNC	6.47E-01	8.47E-05	postive
CLTC	7.08E-01	8.51E-06	postive

PCMT1	6.46E-01	8.52E-05	postive
RG9MTD1	6.46E-01	8.54E-05	postive
ROBLD3	6.46E-01	8.60E-05	postive
NAT5	8.29E-01	8.62E-09	postive
PSMA3	7.07E-01	8.63E-06	postive
DNTTIP1	7.56E-01	8.65E-07	postive
TBCE	7.56E-01	8.65E-07	postive
MGST3	6.46E-01	8.67E-05	postive
LOC402026	6.46E-01	8.71E-05	postive
ZNF167	6.46E-01	8.72E-05	postive
C1QTNF5	-6.46E-01	8.74E-05	negative
LPP	-6.46E-01	8.76E-05	negative
TMEM14C	6.46E-01	8.77E-05	postive
MRPL39	7.07E-01	8.77E-06	postive
HIBCH	6.46E-01	8.80E-05	postive
DUSP12	7.56E-01	8.80E-07	postive
SLC35B1	6.45E-01	8.81E-05	postive
FCN1	-6.45E-01	8.88E-05	negative
LRP1B	7.07E-01	8.91E-06	postive
LEPREL2	-6.45E-01	8.94E-05	negative
DYX1C1	6.45E-01	8.95E-05	postive
ILF2	8.28E-01	8.95E-09	postive
RB1CC1	7.06E-01	8.98E-06	postive
SLC9A6	6.45E-01	9.00E-05	postive
MRPS26	6.45E-01	9.03E-05	postive
RFWD2	7.06E-01	9.14E-06	postive
LOC100128439	-6.44E-01	9.15E-05	negative
RBM4B	6.44E-01	9.17E-05	postive
DDX46	6.44E-01	9.18E-05	postive
PIGY	6.44E-01	9.18E-05	postive
LOC645693	6.44E-01	9.22E-05	postive
RAN	7.55E-01	9.25E-07	postive
PKIB	7.06E-01	9.26E-06	postive
SMARCAL1	7.06E-01	9.27E-06	postive
TRAK2	7.55E-01	9.27E-07	postive
TNFRSF10B	-7.06E-01	9.28E-06	negative
CNN2	-7.55E-01	9.28E-07	negative
CCDC82	6.44E-01	9.32E-05	postive
TMEM145	-7.55E-01	9.32E-07	negative
BHLHE22	6.44E-01	9.34E-05	postive
BLOC1S2	6.44E-01	9.41E-05	postive
GINS3	6.43E-01	9.43E-05	postive
RNF122	-6.43E-01	9.44E-05	negative
FAM44B	6.43E-01	9.46E-05	postive

RCHY1	6.43E-01	9.47E-05	postive
SLC16A3	-7.05E-01	9.47E-06	negative
RNF14	7.54E-01	9.47E-07	postive
CALM2	7.05E-01	9.52E-06	postive
FTSJD1	6.43E-01	9.53E-05	postive
ZC3H15	6.43E-01	9.53E-05	postive
TMEM185A	-6.43E-01	9.56E-05	negative
NRXN2	-6.43E-01	9.61E-05	negative
ARL1	7.05E-01	9.61E-06	postive
NSUN2	7.05E-01	9.61E-06	postive
FAM136A	6.43E-01	9.67E-05	postive
KLHDC5	6.43E-01	9.67E-05	postive
ACTN1	-6.42E-01	9.77E-05	negative
CDC16	6.42E-01	9.78E-05	postive
C2orf47	7.04E-01	9.79E-06	postive
PPCS	7.54E-01	9.79E-07	postive
LOC643779	6.42E-01	9.81E-05	postive
FPR1	-6.42E-01	9.85E-05	negative
DPM1	7.04E-01	9.85E-06	postive
CCT8	6.42E-01	9.86E-05	postive
KIAA0776	7.04E-01	9.89E-06	postive
THAP6	7.04E-01	9.91E-06	postive
NDUFA12	6.42E-01	9.92E-05	postive
